# *DICER-LIKE 5* loss causes thermosensitive male sterility in durum wheat and reveals an AU-rich motif guiding 24-nt phasiRNA biogenesis

**DOI:** 10.1073/pnas.2504349122

**Published:** 2025-07-30

**Authors:** Sébastien Bélanger, Azahara C. Martín, D. Blaine Marchant, Junpeng Zhan, Madison McGregor, Mark Smedley, Sadiye Hayta, Graham Moore, Blake C. Meyers

**Affiliations:** ^a^Donald Danforth Plant Science Center, St. Louis, MO 63132; ^b^Department of Cell and Molecular Sciences, The James Hutton Institute, Dundee, Scotland DD2 5DA, United Kingdom; ^c^John Innes Centre, Norwich Research Park, Norwich NR4 7UH, United Kingdom; ^d^Institute for Sustainable Agriculture-Spanish National Research Council, Córdoba 14004, Spain; ^e^Biology Department, University of Missouri–St. Louis, St. Louis, MO 63121; ^f^National Key Laboratory of Crop Genetic Improvement, Huazhong Agricultural University, Wuhan 430070, China; ^g^Hubei Hongshan Laboratory, Huazhong Agricultural University, Wuhan 430070, China; ^h^The Genome Center, University of California Davis, Davis, CA 95616; ^i^Department of Plant Sciences, University of California Davis, Davis, CA 95616

**Keywords:** wheat, anther, phasiRNA, sterility, Dicer

## Abstract

Reproductive, male-enriched small RNAs are present in flowering plants and animals, yet their role in plants remains underexplored. We generated *dicer-like 5* (*dcl5*) mutants in durum wheat (*Triticum turgidum* ssp. *durum* 2n = 4× = 28; AABB), revealing temperature-sensitive genic male sterility. Loss of *DCL5* depleted premeiotic and meiotic 24-nt phasiRNA production, correlating with sterility under standard growth conditions and partial fertility recovery at higher temperatures. A single functional *DCL5* allele restored complete fertility, presenting a promising alternative to current methods for hybrid production. We demonstrate that premeiotic 24-nt phasiRNA biogenesis is independent of miRNA-mediated cleavage and driven by a conserved motif initiating DCL5 activity. In the *dcl5* mutant developing under sterility-inducing conditions, developmental defects are observed during pollen maturation, rather than at peak 24-nt phasiRNA accumulation in premeiotic and meiotic anthers. Although no visible morphological abnormalities were apparent during early development, single-cell RNA sequencing revealed that *dcl5* mutant cells exhibit transcriptional profiles distinct from those of wild-type cells, when premeiotic 24-nt phasiRNAs are accumulating at the early developmental stage. Finally, the coexpression of *Argonaute* (*AGO1b*, *AGO4a,* and *AGO6*) homeologs in 24-nt phasiRNA-producing cells identifies candidate effectors and suggests a role for 24-nt phasiRNAs in transcriptional gene silencing.

## Significance

To meet rising demand, wheat yields must increase 60% by 2050, even as climate stress threatens productivity. Hybrid vigor is a proven strategy to boost yield but remains underused in self-pollinating cereals due to challenges in pollen control. Current wheat hybrids rely on complex, unstable cytoplasmic male sterility systems. We propose a nuclear-based alternative. In durum wheat, *dcl5* mutants lacking premeiotic and meiotic 24-nt phasiRNAs show temperature-sensitive genic male sterility. A single wild-type allele restores fertility, supporting the feasibility of a *dcl5*-based hybrid system. RNA profiling identified a unique premeiotic 24-nt phasiRNA pathway and coexpression of *ARGONAUTE* homeologs in phasiRNA-producing cells, suggesting a role in transcriptional silencing. This approach could enable reliable, temperature-dependent fertility control in wheat and related crops.

Demand for cereal products is rising with the growing global population, while climate change threatens to reduce crop yield, grain quality, and cultivation areas. Additionally, there is increasing demand and regulatory pressure to reduce agrochemical inputs, which can also impact yield and quality. Exploiting hybrid vigor (or heterosis) can help address these challenges. The use of hybrid varieties takes advantage of this phenomenon, resulting in superior performance compared to nonhybrid plants. This superiority is often seen as higher yields but can also be associated with other desirable traits such as improved quality, better response to fertilizers, better root penetrance, and better rate/duration of grain filling (1, 2). Given the current challenges of fluctuating worldwide environmental conditions, hybrid varieties are of significant importance because they also exhibit greater environmental tolerance, making them suitable for a broader range of locations and environments (3).

Adoption of hybrid varieties in self-pollinated crops, particularly cereals, has previously been limited, with the notable exception of hybrid rice in China (1). This limitation is largely due to the lack of an efficient, cost-effective, and sustainable system for large-scale hybrid seed production. The polyploid nature and complex genetics of cultivated wheat (*Triticum* sp.) have further complicated development of an efficient hybrid wheat production system. Despite reports indicating a 10 to 20% increased yield from the best commercial varieties (4), hybrids currently account for less than 1% of global wheat production. Hybrid seed production systems involve the development of strategies to control pollen production in the female parent through mechanisms inducing male sterility (MS). In wheat, this is largely achieved using chemical hybridization agents (CHAs), cytoplasmic male sterility (CMS), and genic male sterility (GMS) systems (1, 5). CHAs have been used successfully in recent years (1, 2), although environmental impact, health risks, regulatory challenges, and sustainability concerns limit their widespread adoption. The CMS system involves three lines: an MS female parent (the CMS line), a maintainer line to propagate the MS line, and a restorer line carrying one or more fertility restorer genes (6). Despite more than 70 sterilizing cytoplasm types being reported, CMS systems face multiple challenges such as limited line availability, unstable or incomplete male sterility and fertility restoration, and/or hybrid grain quality issues (7–10). In the case of GMS systems, most nuclear male sterility gene mutants or variants are recessive in nature (11). Thus, female GMS line fertility can be restored by any wild-type (WT) accession, offering a wider selection of suitable paternal lines for production of hybrid cereals with superior heterosis. However, a maintainer line is still needed for female male-sterile line propagation. Propagation of GMS lines is therefore costly for large-scale production of hybrids.

An alternative to the previously described systems is the use of environment-sensitive genic male sterility (EGMS) lines. These lines exhibit male sterility under specific environmental conditions but can recover male fertility and propagate via self-pollination when conditions are favorable. This allows the development of a two-line hybrid system. Alternative forms of EGMS systems are photoperiod-sensitive male sterility (PGMS) and temperature-sensitive genic male sterility (TGMS) systems. Rice (*Oryza sativa*) and maize (*Zea mays*) have small RNA (sRNA)-based PGMS and TGMS systems based on perturbations in their reproductive phased small interfering RNAs (phasiRNAs) or regulation factors (12–14). Fertility in PGMS and TGMS lines can be restored by adjusting photoperiod or temperature, creating conditional sterility/fertility and leading to the development of a two-line hybrid system. In rice, EGMS-based hybrids outperform CMS-based hybrids by 5 to 10% in yield, accounting for 30% total hybrid rice in China (7, 12).

Previous work in rice and maize (and subsequently many other plant species) classified reproductive phasiRNAs into either premeiotic, 21-nucleotide (nt) or meiotic, 24-nt phasiRNAs. More recently, a group of premeiotic 24-nt phasiRNAs was identified, accumulating in anthers of barley (*Hordeum vulgare*) and bread wheat (*Triticum aestivum*) (15). Subsequent studies confirmed the presence of such premeiotic phasiRNAs in several other grasses (16, 17). In rice, the biogenesis of reproductive 21-nt phasiRNAs begins with miR2118 directing ARGONAUTE 1d (AGO1d) to cleave phasiRNA precursor (*PHAS* precursors) transcripts. These transcripts are converted into double-stranded RNA (dsRNA) by RNA-DIRECTED RNA POLYMERASE 6 (RDR6), processed into 21-nt sRNA duplexes by DICER-LIKE 4 (DCL4), and loaded onto Argonaute effectors (AGO1d or AGO5c), for posttranscriptional gene silencing (PTGS) (18–21). The meiotic 24-nt phasiRNA pathway involves AGO1d, miR2275, RDR6, and DCL5 (14, 18–21), but their AGO effectors and downstream regulatory mechanisms remain largely unknown. Unlike the meiotic group, premeiotic 24-nt phasiRNAs are unlikely to be triggered by microRNAs (miRNAs), and are presumably loaded onto a different AGO effector (16, 17). While 21-nt phasiRNAs are found in both vegetative and reproductive tissues, so far, 24-nt phasiRNAs have only been observed in male reproductive development.

Recently, Zhang et al. (22) developed *rdr6*, *dcl4*, and *dcl5* mutants, studying how the loss of these genes impacts male fertility in hexaploid bread wheat. They found that loss of *RDR6* resulted in severe consequences, starting with seed germination and continuing throughout plant development and reproduction. Similarly, loss of *DCL4* induced pleiotropic effects in vegetative and reproductive developments, including male sterility. Conversely, loss of *DCL5* led to male sterility alone, with no other developmental defects. While *DCL5* deficiency confers temperature-sensitive genic male sterility in maize (14), no experiments were conducted by Zhang et al. (22) to determine whether environmental conditions could restore male fertility in the bread wheat *dcl5* mutant. In this study, we generated *dcl5* mutants in durum wheat to investigate the molecular and developmental response to environmental conditions. We found that loss of *DCL5* blocks premeiotic and meiotic 24-nt phasiRNA production, inducing a TGMS phenotype with male sterility under standard growth conditions but partial restoration of male fertility at increasing temperatures. RNA profiling of WT and *dcl5* mutants revealed that in fertile *dcl5* anthers, meiotic 24-*PHAS* precursors are activated at the premeiotic stage. Our degradome data, consisting of sRNA-guided cleavage products, demonstrate that premeiotic 24-nt phasiRNA biogenesis does not rely on miRNA-mediated cleavage, suggesting an alternative mechanism for the initiation of dsRNA synthesis by RDR, with the conserved motif playing a potential role in the guiding of DCL5 activity.

## Results

### Loss of *DCL5* Induces Temperature-Sensitive Genic Male Sterility.

In grasses, *DCL5* is required for the biogenesis of 24-nt phasiRNAs (14). Tetraploid durum wheat (*Triticum turgidum* ssp. *durum*; 2n = 4× = 28; AABB) has two *DCL5* copies: *TtDCL5-A1* and *TtDCL5-B1* (23). To study the function of 24-nt phasiRNAs in durum wheat, we selected *dcl5* TILLING mutants with EMS-induced heterozygous stop-gain mutations in *TtDCL5-A1* (Kr4585) and *TtDCL5-B1* (Kr2086) (*SI Appendix*, Fig. S1 and Dataset S1). We used these mutants to develop single (AAbb, Aabb, aabB, and aaBB) and double (aabb) *dcl5* lines.

**Fig. 1.**
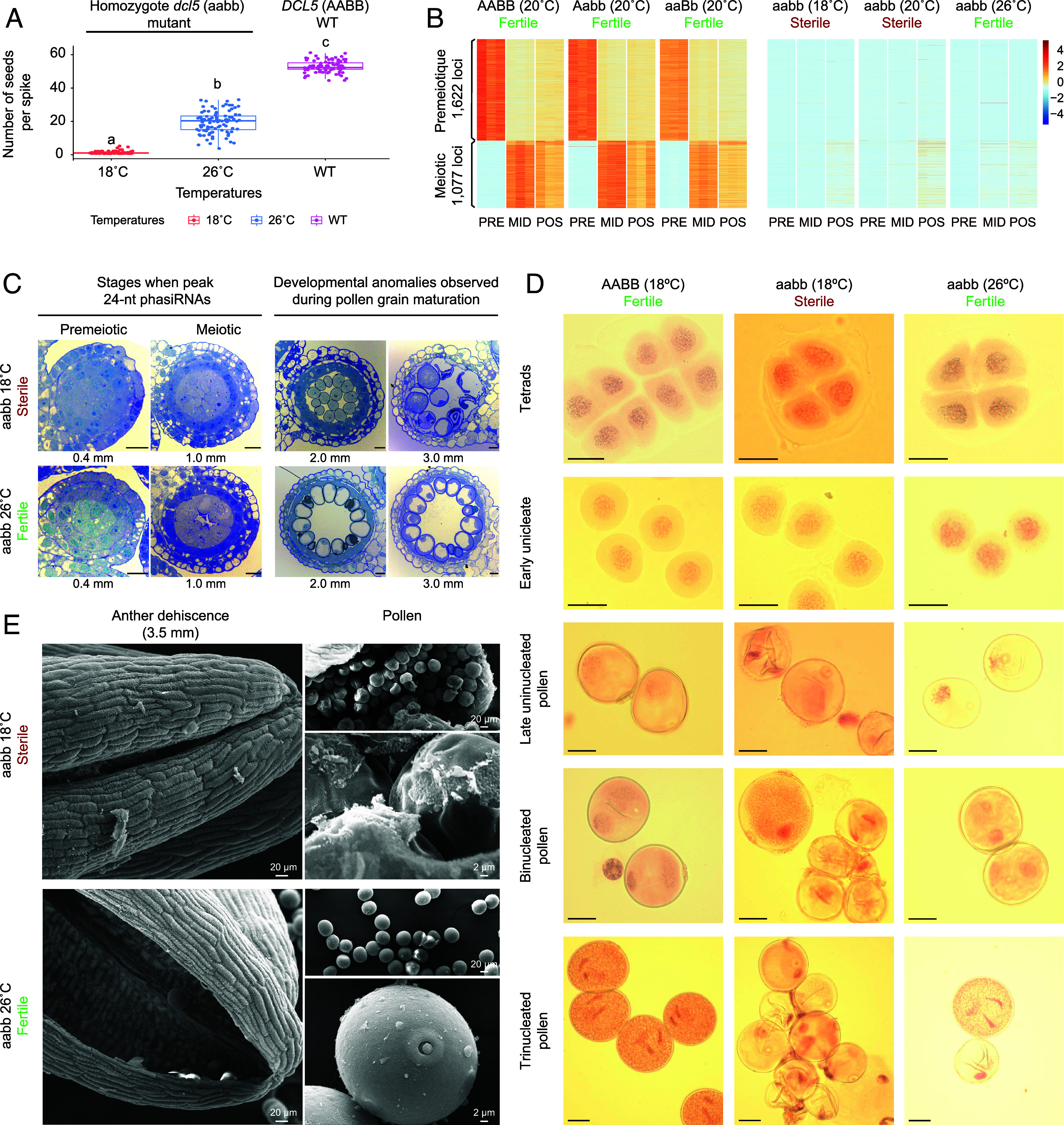
Loss of DCL5 controls pollen production and induces a temperature-sensitive genic male sterile phenotype. (*A*) Number of seeds retrieved in spikes of the five most productive tillers. Letters above the box plot indicate that all tested comparisons were significant. (*B*) The relative abundance of 24-nt phasiRNAs sequenced in triplicates of premeiotic (PRE), meiotic (MID), and postmeiotic (PST) anthers of WT (AABB), the mutant expressing a single functional allele (Aabb and aabB), and the homozygous double mutant (aabb). (*C*) Light microscopy images show cross-sections of anthers at four stages of development in the *dcl5* mutants under restrictive conditions (*Top*) and permissive conditions (*Bottom*). (Scale bar, 20 µm.) (*D*) Microgametogenesis of WT and dcl5 (aabb) mutants at sterile (18 °C) and fertile (26 °C) temperature conditions. (Scale bar, 20 μm.) (*E*) SEM was conducted on mature anthers from *dcl5* mutants grown under restrictive (*Top*) and permissive (*Bottom*) conditions. Scale bars are indicated in micrographs.

We grew WT and the different *dcl5* genotypes under standard conditions (20 °C-day and 15 °C-night, with a 16-h photoperiod) to examine the impact of *DCL5* loss-of-function on vegetative and reproductive developments. A single functional *DCL5* allele (Aabb or aabB) was enough to maintain normal fertility, as evidenced by full seed set and no vegetative developmental defects (*SI Appendix*, Fig. S2*A*). However, while the homozygous *dcl5* double mutant (aabb) grew normally, no seeds were recovered, indicating reproductive failure (Fig. 1*A* and *SI Appendix*, Fig. S2*B*). This reproductive failure was confirmed to be specific to male development, as using this *dcl5* (aabb) mutant as the pollen recipient in backcrosses with WT pollen resulted in a complete seed set.

To check whether changes in the environmental conditions could restore male fertility in the double *dcl5* mutant, we grew the mutant under different temperatures, at increments of 2 °C between 18 °C and 26 °C (Fig. 1*A* and *SI Appendix*, Fig. S2*C*). At 18 °C and 20 °C, corresponding to standard growth temperatures for durum wheat, seed recovery was minimal or null. However, fertility was partially restored at daytime temperatures of 22 °C (17 °C-night) and above, with the optimal temperature being 26 °C-day (19 °C-night), where one-third of the normal WT seed set level was recovered, demonstrating the temperature-sensitive genic male-sterile (TGMS) nature of the *dcl5* double mutant (aabb) (Fig. 1*A* and *SI Appendix*, Fig. S2*C*). We also tested the response of the *dcl5* mutant to shorter (14 h) and longer (18 h) day photoperiods at 18 °C and 26 °C but found no photoperiod-sensitive phenotype (*SI Appendix*, Fig. S2*D*). These results suggest the potential for using the 24-nt phasiRNA pathway to control pollen production via the *DCL5* gene, developing a TGMS line for a two-line hybrid production system in wheat.

### Loss of *DCL5* Depletes Premeiotic and Meiotic 24-nt phasiRNAs.

We performed sRNA sequencing on samples from premeiotic, meiotic, and postmeiotic anthers of the WT (AABB), mutants with a single functional allele (Aabb and aabB), and the homozygous double mutant (aabb). In the durum wheat genome, 2,699 loci expressed 24-nt phasiRNAs, with 1,622 loci active in the premeiotic stage and 1,077 loci active in meiotic/postmeiotic stages (Fig. 1*B* and Dataset S2). Mutants with one functional *DCL5* allele (Aabb or aabB) showed normal 24-nt phasiRNA accumulation and maintained complete fertility (Fig. 1*B* and *SI Appendix*, Fig. S2*B*). In contrast, the homozygous double mutant (aabb) completely lacked 24-nt phasiRNAs, indicating the role of *DCL5* in the biogenesis of both premeiotic and meiotic groups (Fig. 1*B*). Loss of *DCL5* disrupted anther 24-nt phasiRNA production under temperatures inducing both male sterility (18 °C and 20 °C) and partial fertility (22 °C) (Fig. 1*B*). However, loss of *DCL5* did not affect 21-nt reproductive phasiRNA production (*SI Appendix*, Fig. S3 and Dataset S2). These results demonstrate that *DCL5* disruption impedes 24-nt phasiRNA biosynthesis in homozygous *dcl5* plants, leading to male sterility, but with no corresponding effect on female fertility. Collectively, these results further demonstrate that homozygous *dcl5* plants could be valuable as the (male sterile) female parent for F1 hybrid seed production. Hybrid offspring heterozygous for the *DCL5* loci will regain 24-nt phasiRNA synthesis, restoring male fertility and seed production.

### Developmental Defects Are Observed during Pollen Maturation Rather than at the Peak Accumulation of 24-nt phasiRNAs.

To determine whether developmental defects arise in anthers with accumulation of 24-nt phasiRNAs, we conducted a cytological analysis of anthers at thirteen developmental stages in both WT and the *dcl5* mutant (aabb), under sterility-inducing (18 °C; restrictive) and fertility-inducing (26 °C; permissive) temperatures (*SI Appendix*, Fig. S4). Compared to WT anthers, *dcl5* mutants showed no notable differences at stages when 24-nt phasiRNAs typically accumulate, specifically premeiosis (0.2 to 0.4 mm) and early meiosis (0.8 to 1.0 mm), despite slower development at 18 °C (Fig. 1*C* and *SI Appendix*, Fig. S4). Meiosis completed normally in mutants grown under restrictive conditions, evidenced by normal formation of microspore tetrads, the final product of male meiosis (Fig. 1*D* and *SI Appendix*, Fig. S5). However, sterile anthers formed a thick, persistent tapetum that did not shrink at the microspore stage, displaying a “Swiss cheese” appearance due to cell holes (Fig. 1*C* and *SI Appendix*, Fig. S4). Additionally, the sterile mutant had abnormal pollen shape, size, and distribution before anther dehiscence, unlike the fertile mutant (Fig. 1*C* and *SI Appendix*, Fig. S4). In contrast, both WT and mutant individuals developed under permissive conditions formed normal tapetal cells and pollen grains. To further investigate pollen development and pinpoint the stage at which developmental issues begin to arise, we closely monitored microspore progression from the tetrad stage to mature trinucleate pollen (Fig. 1*D*). Our observations revealed that abnormal development in the *dcl5* (aabb) mutant, under sterility-inducing conditions at 18 °C, occurred specifically during the late uninucleate stage, following the appearance of the germinal pore. In summary, our cytological analysis reveals that developmental defects do not temporally align with the peak accumulation of 24-nt phasiRNAs at premeiotic and meiotic stages. Instead, these defects are observable during pollen grain maturation.

### Dysfunctional Pollen Development and Anther Dehiscence Are Contributing Factors to Male Sterility.

Building on observations from LM imaging, we used transmission electron microscopy (TEM) to examine the ultrastructure of tapetum and pollen cells in anthers ranging from 1.8 to 3.5 mm (*SI Appendix*, Fig. S6). Developmental rate varied between mutants at 18 °C and 26 °C, with 2.0 mm anthers at 18 °C corresponding to 1.8 mm anthers at 26 °C. At the restrictive (18 °C) temperature, 2.0 mm *dcl5* anthers showed a thick Swiss cheese tapetum with sparse, small, nonuniformly distributed Ubisch bodies at the periphery of the cell wall closest to the germinal cells, unlike 2.0 mm *dcl5* anthers at the permissive (26 °C) temperature (*SI Appendix*, Fig. S6). The sterile mutant displayed less intimate contact between the tapetum, Ubisch bodies, and pollen compared to the fertile mutant, and the pollen cell walls were thinner (*SI Appendix*, Fig. S6). At later stages, under restrictive temperature, additional differences in pollen cell wall thickness were noted: The inner side was thicker and the outer side thinner in 3.0 to 3.5 mm anthers, leading to rupture of some pollen grains (*SI Appendix*, Fig. S6). Under permissive temperature, the thickness of the mutant pollen cell wall was normal (*SI Appendix*, Fig. S6). These results suggest that loss of *DCL5* affects tapetal cell development or metabolic activity, impacting Ubisch body production and pollen cell wall development.

Although most pollen grains in the sterile mutant showed abnormal phenotypes, some appeared relatively normal and potentially fertile (Fig. 1*D*). To investigate this further, we used scanning electron microscopy (SEM) to examine anther and pollen grain surfaces. Under restrictive conditions, incomplete anther dehiscence impeded pollen release. Manually rupturing the anther revealed wrinkled and agglutinated pollen grains adhering both to each other and the tapetum (Fig. 1*E*). Conversely, anthers developing under permissive conditions displayed normal dehiscence, releasing uniform pollen grains (Fig. 1*E*). Overall, TEM and SEM observations highlighted anomalies at interfaces between tapetum, Ubisch body, and pollen, including changes in the cell wall matrix that promote cohesion between pollen grains, and between the tapetum and pollen.

### Premeiotic 24-nt phasiRNA Biogenesis Is Not Triggered by miRNA-Mediated Cleavage.

**Fig. 2.**
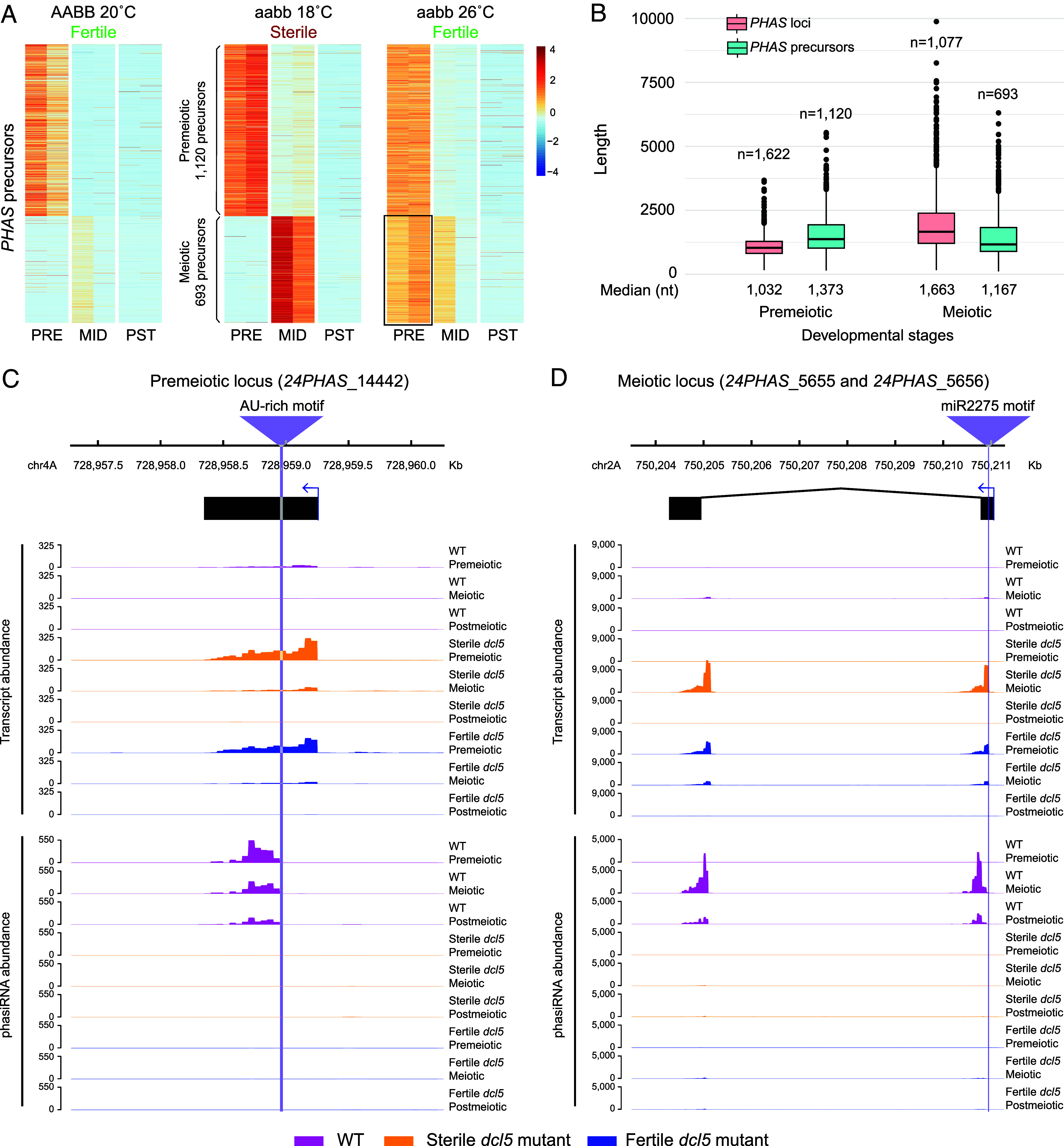
Premeiotic 24-nt phasiRNA biogenesis is not triggered by miRNA-mediated cleavage, but the conserved motif is important for Dicer-like processing. (*A*) The relative abundance of 24-*PHAS* precursors in duplicates of premeiotic (PRE), meiotic (MID), and postmeiotic (PST) anthers of WT (AABB) and homozygous *dcl5* mutants (aabb) developing under conditions inducing sterility and partial fertility. (*B*) Box plots of *PHAS* loci and precursors indicate a similar length distribution. (*C* and *D*) Distribution of mRNA, sRNA, and nanoPARE sequencing reads mapped to premeiotic (*Left*) and meiotic (*Right*) *PHAS* loci in WT (magenta) and *dcl5* anther samples under restrictive (orange) and permissive (blue) conditions. Premeiotic and meiotic motifs are positioned at the vertical purple lines.

We performed mRNA and nanoPARE (nano-Parallel Analysis of RNA 5’ Ends) sequencing on anther samples from WT and the *dcl5* (aabb) mutant, grown under both sterility-inducing (18 °C; restrictive) and fertility-inducing (26 °C; permissive) temperatures. In WT, 24-*PHAS* precursors were efficiently processed into 24-nt phasiRNAs, preventing the identification of many precursor transcripts (Fig. 2*A*). Conversely, in *dcl5* mutants, 24-*PHAS* precursors accumulated in anthers as they were not converted into 24-nt phasiRNAs (Fig. 2*A*), allowing us to identify 1,813 *PHAS* precursors: 1,120 of 1,622 premeiotic loci and 693 of 1,077 meiotic loci. Premeiotic and meiotic *PHAS* precursor lengths were similar (Fig. 2*B*). Overall, reads mapping to identified 24-*PHAS* sequences represented 0.89% of total transcript reads. *PHAS* precursor accumulation coincided with the accumulation of corresponding premeiotic and meiotic 24-nt phasiRNAs in the *dcl5* mutant under restrictive temperature (Fig. 2*A*). Notably, in the mutant under permissive conditions, *PHAS* precursors for meiotic 24-nt phasiRNAs accumulated at the premeiotic stage (Fig. 2*A*).

**Fig. 3.**
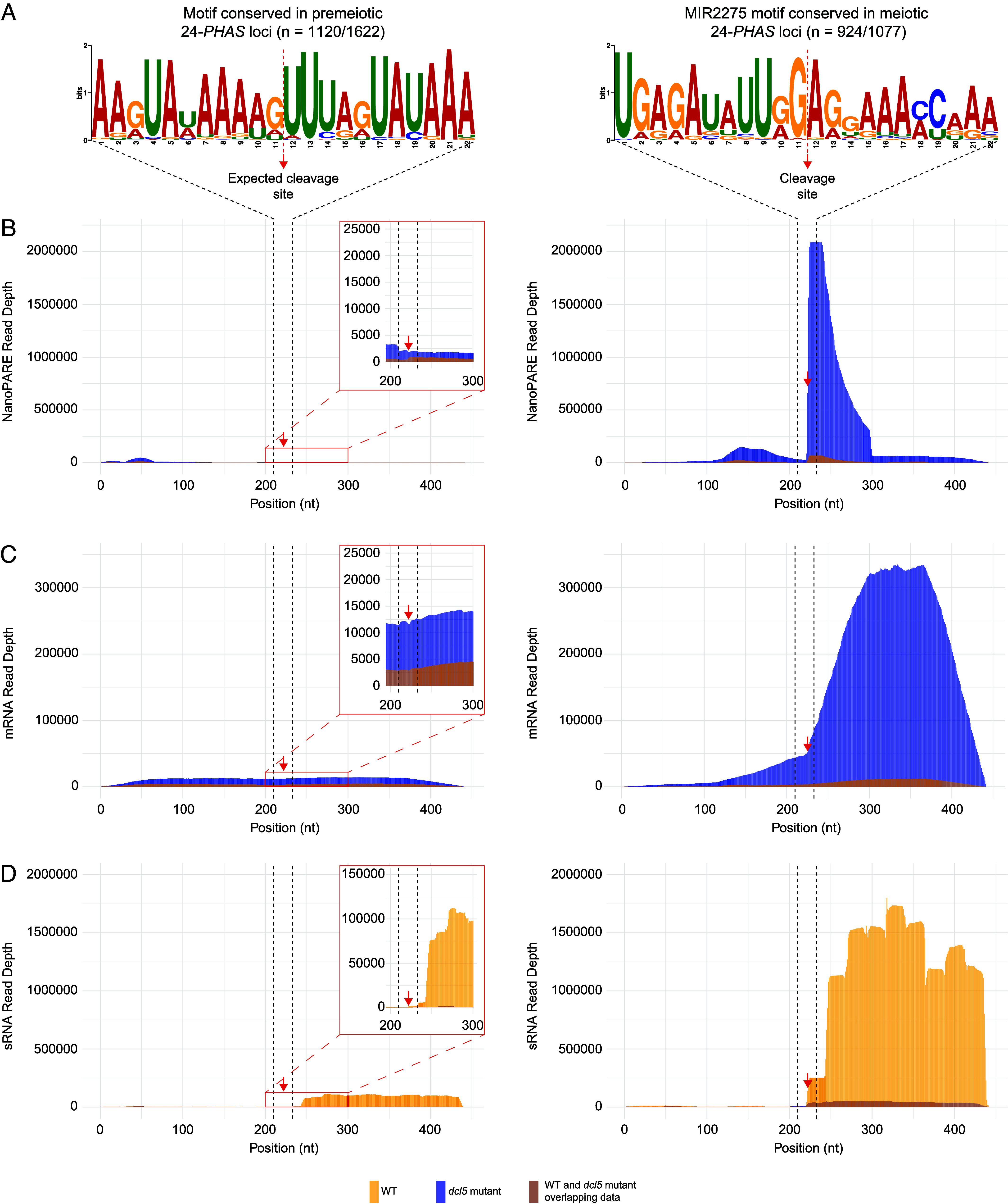
Genome-wide analysis of degradome, mRNA, and sRNA confirms the unique biogenesis mechanism of premeiotic 24-phasiRNAs. (*A*) Conserved motifs enriched in premeiotic and meiotic in 24-*PHAS* loci, as indicated. (*B*–*D*) Mapping of nanoPARE (*B*), mRNA (*C*), and sRNA (*D*) reads across these motifs and their flanking regions (200 nucleotides upstream and downstream). *Inset* panels with adjusted y-axis scales focus on reads mapped at or near the AU-rich motif. Red dashed lines and arrows indicate predicted cleavage sites at the premeiotic and meiotic motifs, respectively, while black dashed lines denote motif boundaries. RNA libraries were prepared in two biological replicates for three developmental stages from WT (orange) and *dcl5* (blue) lines, grown under both sterile and fertile conditions. The data from the replicates were combined to generate the tracks.

Previous studies identified an adenine and uracil (AU)-rich motif among premeiotic 24-*PHAS* precursors and suggested that these were not triggered by miRNA-mediated cleavage, but by an unknown mechanism (16, 17). To verify this, we analyzed read mapping on premeiotic and meiotic *PHAS* loci. In WT, no read accumulation on *PHAS* precursors was observed, but abundant 24-nt phasiRNAs were present (Fig. 2 *C* and *D*). In mutants grown under both restrictive and permissive conditions, abundant reads aligned to *PHAS* precursors, but no 24-nt phasiRNA production was observed (Fig. 2 *C* and *D*). Thus, sequencing data from WT and *dcl5* mutants provided complementary results for 24-nt phasiRNA biogenesis. In mutants, mRNA and nanoPARE reads accumulated at the miR2275 target motif and downstream, indicating cleavage of the meiotic *PHAS* precursor without 24-nt phasiRNA production (Fig. 2*D*). In WT and mutants, nanoPARE data confirmed miR2275-triggered cleavage of the *PHAS* precursor at the miR2275 motif for meiotic *PHAS* precursors, with phasiRNAs accumulating from the 3’ end of the miR2275 motif in WT (Fig. 2*D*). For premeiotic 24-*PHAS* loci, *dcl5* mutants showed mRNA and nanoPARE read accumulation across the entire precursor, indicating no cleavage of the premeiotic *PHAS* precursor. Interestingly, in WT, sRNA reads accumulated from the conserved AU-rich motif and downstream (Fig. 2*D*), with degradome analysis failing to identify an sRNA responsible for premeiotic *PHAS* precursor cleavage at the conserved motif. To validate these observations, we analyzed genome-wide nanoPARE, mRNA, and sRNA read mapping across premeiotic (1,120/1,622; 69.1%) and meiotic (924/1,077; 85.8%) 24-*PHAS* loci containing a conserved motif using data generated from WT and *dcl5* lines (Fig. 3). As shown in Fig. 3*B*, meiotic loci (right panel) exhibited a strong nanoPARE cleavage signal specifically at the miR2275 motif, whereas no cleavage signal was detected at the AU-rich motif in premeiotic loci (left panel). Notably, mRNA read accumulation was observed downstream of the motif in meiotic 24-*PHAS* loci but spanned the entire region in premeiotic loci (Fig. 3*C*). In both groups, abundant 24-nt phasiRNAs accumulated downstream of these motifs. Curiously, the premeiotic phasiRNAs did not initiate at the midpoint of the AU-rich motif, consistent with the absence of a cleavage pattern, but 12- to 24-nt 3’ of this point (Fig. 3*D*). Despite this seemingly less precise initiation, inspection of example 24-nt premeiotic loci demonstrated clear phasing (*SI Appendix*, Fig. S7). Together, these findings indicate that the biogenesis of premeiotic 24-nt phasiRNAs is independent of miRNA triggering, suggesting that an alternative mechanism recruits the RDR protein for dsRNA generation. Additionally, the conserved motif appears essential for DCL5 recognition and subsequent phasiRNA production in premeiotic loci, but in a manner distinct from miRNA-triggered phasiRNAs.

### Unseen Differences: *dcl5* Cells Are Transcriptionally Distinct from WT at the Premeiotic Stage.

**Fig. 4.**
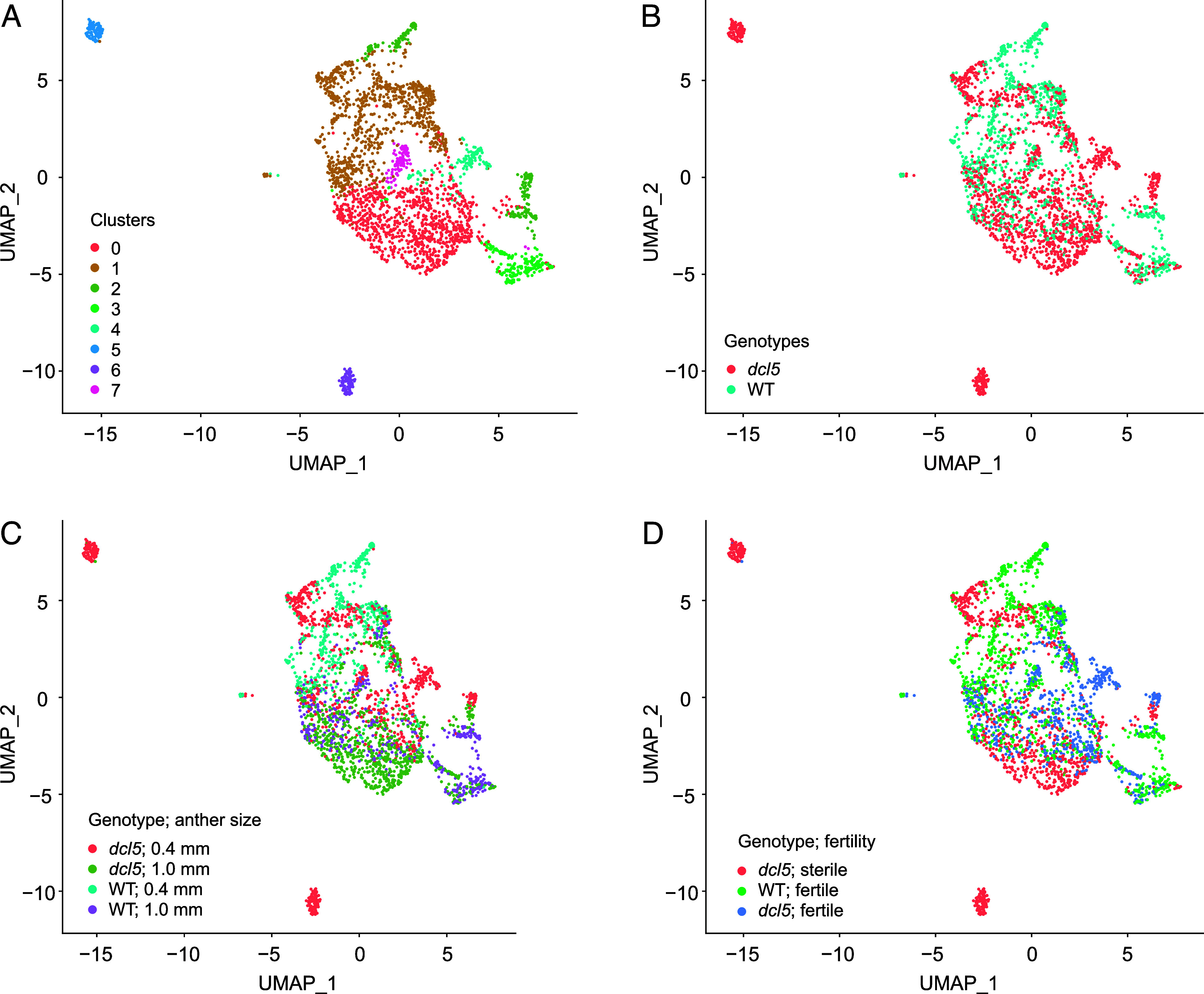
Clustering analysis of scRNA-seq data from dcl5 and WT cells. UMAP clustering of 2,758 cells reveals eight distinct clusters (*A*) representing *dcl5* and WT genotypes (*B*) from 0.4 mm premeiotic and 1.0 mm meiotic durum wheat anthers (*C*) categorized by their sterile or fertile status (*D*). The UMAP plots show three clusters (Clusters 4, 5, and 6) exclusively composed of *dcl5* cells from 0.4 mm anthers, along with two cell clusters from the sterile anther and one from the fertile anther.

To investigate early transcriptional changes in *dcl5* mutants, we performed single-cell RNA-sequencing (scRNA-seq) of anthers at 0.4 mm and 1.0 mm stages, corresponding to premeiotic and meiotic 24-nt phasiRNA accumulation. Analysis was conducted under permissive and restrictive temperature conditions, with WT as control. Quality control was applied to 3,456 single-cell samples, and 2,758 cells passed preprocessing. The dataset revealed 8 clusters: Most clusters were evenly represented across genotypes and anther stages (Fig. 4); however, three clusters were exclusively represented by *dcl5* cells from 0.4 mm anthers (Clusters 4, 5, and 6), indicating that these *dcl5* cells were transcriptionally distinct from WT during early development when premeiotic 24-nt phasiRNAs are accumulating. In the absence of wheat-specific markers, we used maize cell-specific gene markers (24) to characterize cell clusters, but were unable to annotate specific cell types, seemingly due to the many allopolyploid genome paralogs.

Overall, as previously observed in whole anthers, 21-*PHAS* and 24-*PHAS* transcripts make up a small fraction of those totally expressed, except in a small number of cells with greater abundance (*SI Appendix*, Fig. S8). As expected, in both WT and *dcl5* lines, more 0.4 mm premeiotic cells express abundant 21-*PHAS* transcripts than do 1.0 mm meiotic cells, although this is less obvious in the sterile *dcl5* cells (*SI Appendix*, Fig. S8*A*). Notably, cells from fertile *dcl5* anthers at the 0.4 mm premeiotic stage express a much higher proportion of 24-*PHAS* transcripts and in a greater number of cells (*SI Appendix*, Fig. S8*B*), seemingly due to the expression of meiotic 24-*PHAS* precursors at the premeiotic stage, as observed in whole anthers (Fig. 2*A*). However, the proportion of 24-*PHAS* transcripts in *dcl5* sterile cells is much lower, even compared to WT.

In maize, four basic helix–loop–helix (bHLH) transcription factors (*MS23*, *MS32*, *bHLH122*, and *bHLH51*) sequentially regulate the expression of 24-*PHAS* precursors and *DCL5* in tapetal cells (25). Assuming these genes regulate both premeiotic and meiotic 24-nt phasiRNA pathways and that their function is conserved in wheat, we used them as markers, along with *DCL5*, to identify putative tapetal cells or other cells involved in anther 24-nt phasiRNA biogenesis. Analysis revealed that wheat homologs of *DCL5*, *MS23,* and *bHLH51* were expressed in sampled cells, allowing us to subset 952 putative tapetal cells (or their precursor cells in 0.4 mm anthers) for studying sRNA pathway genes (*SI Appendix*, Fig. S9). Most *RDR* and *DCL* genes associated with phasiRNA biogenesis were undetectable, except for *DCL5*, which was highly expressed in a subset of tapetal cells but absent in nontapetal cells (*SI Appendix*, Fig. S9*A*). The two *DCL5* homeologs were highly expressed in premeiotic WT cells but mainly absent in meiotic WT cells and throughout the *dcl5* cells (*SI Appendix*, Fig. S9*B*), consistent with previous whole anther observations (17). We also observed coexpression of specific *AGO* genes (including *AGO1b*, *AGO4a*, and *AGO6*) in tapetal cells (*SI Appendix*, Fig. S9*A*). The *AGO1b* gene was highly expressed in tapetal cells, while absent in nontapetal cells (*SI Appendix*, Fig. S9*A*). In WT, *AGO1b* was broadly and highly expressed in premeiotic cells, with levels declining sharply in meiotic tapetal cells (*SI Appendix*, Fig. S9*B*). Although *AGO1b* expression was lower and narrower, it followed a similar pattern in sterile and fertile *dcl5* mutant cells. Interestingly, homeologs of *AGO4a* and *AGO6* exhibited distinct expression patterns: One copy of each gene was broadly and highly expressed in tapetal cells, while the other copy had higher expression in nontapetal cells (*SI Appendix*, Fig. S9*A*). When focusing on tapetal cells, although the fraction of cells expressing *AGO4a* and *AGO6* differed between fertile and sterile *dcl5* mutants, these genes were present in premeiotic cells but less abundant and only present in a small fraction of tapetal cells at the meiotic stage (*SI Appendix*, Fig. S9*B*). In contrast, *AGO4a* and *AGO6* were highly abundant in WT tapetal cells during premeiotic and meiotic stages. The above observations suggest that cells likely involved in the biogenesis and function of 24-nt phasiRNA are expressing specific *AGO* homeologs, thereby narrowing down the pool of candidate AGO effectors associated with 24-nt phasiRNAs.

## Discussion

Recent research has examined the role of *DCL5* in cereal male reproduction. For example, in bread wheat, a *dcl5* triple mutant was recently reported with male sterility under normal photoperiod and temperature conditions (22). This sterility was characterized by anomalies at the uninucleate microspore and subsequent stages, along with a lack of meiotic 24-nt phasiRNAs in early postmeiotic anthers at the microspore tetrad stage (aligning with our own observations); however, no analysis of premeiotic or meiotic anthers was reported (22). In maize, the *dcl5* mutant shows temperature sensitivity during meiosis, with arrest in tapetal development observed at this and subsequent stages, coupled with a lack of meiotic 24-nt phasiRNAs (14). Differences in temperature-dependent regulation of sterility and fertility developmental transitions in maize and durum wheat *dcl5* mutants are intriguing. The maize *dcl5* mutant exhibits male sterility under typical high growth temperatures (28 °C), with lower temperatures (≤26 °C) restoring fertility. In contrast, the durum wheat *dcl5* mutant shows sterility at normal low temperatures (≤20 °C), with higher temperatures (≥22 °C) restoring fertility. These differences suggest a potential role for 24-nt phasiRNAs in mediating species adaptation to ecological environments, maintaining fertility in response to temperature fluctuations.

We have demonstrated that loss of *DCL5* inhibits the production of both premeiotic and meiotic 24-nt phasiRNAs, as occurs in the *dcl5* maize mutant (16), but no phenotype was observed before the completion of meiotic division. This delayed phenotype is intriguing since 24-nt phasiRNAs—abundant in early cell fate specification and meiosis—are the predominant class of 24-nt sRNAs in wheat anthers (15). Single-cell RNA-sequencing identified three distinct clusters unique to *dcl5* cells from 0.4 mm anthers, revealing transcriptional divergence from WT during early development when premeiotic 24-nt phasiRNAs accumulate, despite no phenotypic differences. This suggests that premeiotic phasiRNAs may set up a specific cell type such as the tapetum for subsequent roles, although the momentum of anther development and cellular differentiation is sufficient to carry all anther cell layers through meiosis. In the absence of *DCL5*, 24-*PHAS* precursors accumulate as expected, as they are not processed into 24-nt phasiRNAs. In *dcl5* sterile anthers, *PHAS* precursor accumulation correlates with the two groups of 24-nt phasiRNAs (premeiotic and meiotic) described in WT. Notably, in *dcl5* anthers that have recovered fertility, *PHAS* precursors are actively transcribed early in the premeiotic stage, resulting in premature accumulation of meiotic *PHAS* precursors. Taken together, these observations are intriguing. First, the complete absence of 24-nt phasiRNAs in *dcl5* lines—both in sterile and fertile anthers—suggests that fertility restoration may occur independently of 24-nt phasiRNA production. Second, the temporal shift in *PHAS* precursor accumulation in mutants recovering fertility implies that temperature can reprogram transcriptional timing, revealing latent redundancy in phasiRNA biogenesis pathways that underlie developmental and reproductive plasticity. These findings invite further investigation into how small RNA pathways, including 24-nt phasiRNAs, may have evolved to support fertility across diverse or fluctuating environments, or in pathways that are impacted by environmental conditions.

To date, the AGO effectors responsible for 24-nt phasiRNA function remain unidentified. We previously reported that *AGO4a*/*c* and *AGO6* genes are coexpressed with premeiotic and meiotic 24-nt phasiRNAs, respectively, and that this expression pattern is conserved across seven species of grass (17). Notably, in this study, we found that both *AGO4a* and *AGO6* homeologs are coexpressed in cells producing 24-nt phasiRNAs. In *Arabidopsis*, AGO4/6/9-family proteins function in RNA-directed DNA methylation (RdDM), a pathway guided by 24-nt siRNAs (26). Based on these findings, we propose that 24-nt phasiRNAs may interact with AGO4a and AGO6 to mediate transcriptional or chromatin-level regulation during anther development. Whether this regulation involves canonical RdDM or other small RNA-directed pathways remains to be determined, particularly in the context of temperature-sensitive fertility.

Our previous work described that premeiotic 24-nt phasiRNAs differ from those abundant during meiosis by a conserved, AU-rich motif in premeiotic 24-*PHAS* precursors, suggesting that they are not triggered by miRNA-mediated cleavage but by an unidentified mechanism (16, 17). Here, comparative RNA profiling (sRNA, mRNA, and nanoPARE) of WT and *dcl5* mutants clearly demonstrates that 1) premeiotic phasiRNA biogenesis may be initiated not by miRNA-mediated cleavage but by an unknown mechanism that recruits RDR proteins for dsRNA synthesis, and 2) the AU-rich motif is important for guiding DCL5 activity. Plant phasiRNA production is conventionally thought to be initiated by miRNA-triggered cleavage of target RNA followed by RDR6 and DCL4/5 recruitment, to produce 21-nt or 24-nt phasiRNAs in vegetative or reproductive tissues. Recently, however, our group demonstrated that DCL4 can directly process a dsRNA transgene into 21-nt phasiRNAs, bypassing the steps of miRNA-directed cleavage and dsRNA production, revealing plasticity in phasiRNA biogenesis (27). In conclusion, our study prompts further questions about mechanisms of premeiotic phasiRNA biogenesis: Does RDR6 independently recognize these *PHAS* precursors, or does it rely on an RNA-binding protein for dsRNA initiation? Does DCL5 (or a partner protein) identify the AU-rich motif in the *PHAS* precursor to initiate phasiRNA production, rather than acting via AGO cleavage and uncapped ends?

## Materials and Methods

### Genetic Stock Development, Plant Growth Conditions, and Tissue Harvesting.

Tetraploid wheat *dcl5* TILLING mutants were derived from the durum wheat cultivar “Kronos” (*Triticum turgidum* ssp. *durum* 2n = 4× = 28; AABB), which also served as the WT control. TILLING lines, Kronos4585 (Kr4585) and Kronos2086 (Kr2086), were identified from the Ensembl Plants database (28) and found to have EMS-induced heterozygous mutations in *TtDCL5-A1* (TRITD1Av1G103650) and *TtDCL5-B1* (TRITD1Bv1G104440), respectively. These are stop-gain mutations (Variant IDs: Kronos4585.chr1A.289335805 in *TtDCL5-A1* and Kronos2086.chr1B.322412735 in *TtDCL5-B1*), generating premature stop codons in both homeologs. Homozygous *Ttdcl5-A1* and *Ttdcl5-B1* single mutants were backcrossed with WT Kronos for three generations to eliminate background mutations. The resulting homozygous single mutants were crossed, and the segregating progeny were genotyped using mutation-specific KASP markers to select single (AAbb, Aabb, aabB, and aaBB) and double (aabb) *Ttdcl5* mutants. KASP primer sequences are shown in Dataset S1. Allele-specific primers were synthesized with standard FAM or VIC compatible tails at their 5’ ends (FAM tail: 5’ GAAGGTGACCAAGTTCATGCT 3’; VIC tail: 5’GAAGGTCGGAGTCAACGGATT 3’).

Under normal conditions, the selected mutants were grown in a growth chamber at 20 °C/18 °C day/night, with 16 h of light and 8 h of darkness, and 50% relative humidity. To restore fertility, homozygous double mutant and WT plants were cultivated in a growth chamber at 5 day/night temperatures (18 °C/15 °C, 20 °C/17 °C, 22 °C/17 °C, 24 °C/17 °C, and 26 °C/19 °C) maintaining a 16-h photoperiod and 50% humidity. We counted seeds from the five most productive tillers and performed pairwise comparisons using the “t_test” function from the “ggpubr” and “rstatix” R packages, with *P*-values adjusted by the Bonferroni method.

For cytological and RNA experiments, anthers were dissected using a stereomicroscope (Mantis Elite-Cam HD, Vision Engineering). Anthers ranging from 0.2 to 3.5 mm in length were collected for histological analysis, with 10 anthers per stage. These anthers were promptly fixed with aldehyde as described in the section below. For RNA experiments, anthers at premeiotic (0.2 to 0.4 mm), meiotic (0.8 to 1.0 mm), and postmeiotic (1.8 to 2.0 mm) stages were used. Each stage involved collecting 50, 30, and 15 anthers per sample, respectively, in triplicate. Postdissection, samples were immediately frozen in liquid nitrogen and stored at −80 °C until RNA isolation.

### Tissue Preparation for Light and Electron Microscopy Experiments.

To perform light (LM) and electron (EM) microscopy on the same sample, without resampling, we prepared samples following the method of Bélanger et al. (29) with modifications. Freshly harvested anthers were fixed overnight in 2% [v/v] paraformaldehyde, 2% [v/v] glutaraldehyde, and 0.1% [v/v] Tween20 in 0.1 M sodium cacodylate buffer (pH 7.4). For heavy metal staining, samples were washed (2 × 30 min in water) and transferred to 1.5% [v/v] OsO_4_ buffered in 0.1 M sodium cacodylate buffer for 3 h. Samples were then washed (2 × 60 min in water), incubated in 1% [w/v] aqueous uranyl acetate at 4 °C overnight, heated to 50 °C for 2 h, and washed again (2 × 60 min in water). Prior to infiltration and embedding, samples underwent graded dehydration in cold acetone (30, 50, 70, 80, 90, 100, and 100% [v/v]) at 4 °C for 30 min each. Dehydrated samples were then exchanged twice in 100% propylene oxide (#20401; EMS, Hatfield, PA) for 30 min each. Samples were then infiltrated with a graded series of Quetol (#14640; EMS, Hatfield, PA) in propylene oxide (25, 50, 75, and 100% [v/v]) without DMP-30 at room temperature for 24 h each, using a rocking platform to enhance resin infiltration. Three overnight exchanges in 100% resin with DMP-30 followed. Finally, samples were embedded in fresh 100% Quetol with DMP-30 in flat embedding molds (#70900; EMS, Hatfield, PA) and polymerized at 60 °C for 48 h.

### LM and TEM Image Acquisition.

Samples for LM were sectioned at 500 nm using the Leica Ultracut UCT (Leica Microsystems Inc., Wetzlar, Germany) and stained with Epoxy Tissue Stain (#14950; EMS, Hatfield, PA). Sections were observed using a Leica DM 750 microscope, and images were captured using a Leica ICC50 HD camera and Leica Acquire v2.0 software (Leica Microsystems Inc., Wetzlar, Germany). Image analysis was performed with ImageJ (30). LM results guided the selection of samples for TEM imaging.

Selected samples were sectioned at 70 nm using the Leica Ultracut UCT, mounted on formvar/carbon film on slotted gold grids (#FCF2010-Au-SB; EMS, Hatfield, PA), and stained with either Reynolds’ lead citrate (31) or Sato’s lead solution (32) for 7 min. Grids were then rinsed with degassed double-distilled water and air dried. Sections were imaged with a Thermo Scientific Talos L120C TEM at 120 kV. High-resolution images covering a large area were acquired with a Ceta 16 M CMOS 4 k × 4 k camera and stitched together with Maps 3.16 software.

### Tissue Preparation and SEM Image Acquisition.

Anthers at the predehiscence stage were dissected, fixed, and stained with heavy metal as previously described. The samples were dehydrated in an ethanol series, dried in a Tousimis Samdri-780A critical point dryer, and mounted on aluminum stubs with double-stick carbon adhesive tabs. They were then Au/Pd coated in a Tousimis Samsputter-2a. Images were acquired at 500× and 5,000× magnification using a Zeiss Evo10 SEM at the Biology Imaging Facility of Washington University (St. Louis, MO).

### Tissue Preparation and Imaging of Male Meiocytes.

Anthers from WT and *dcl5* (aabb) plants grown under sterile-inducing (18 °C) or fertile-inducing (26 °C) temperatures were dissected. Anthers were fixed in ethanol:acetic acid (3:1) and stained with Schiff’s reagent (Feulgen method) for microgametogenesis or aceto-carmine for macrogametogenesis, enabling visualization of meiocytes under light microscopy.

### RNA Isolation, Library Construction, and Sequencing.

Total RNA was isolated using the TRI Reagent (Sigma-Aldrich, St. Louis) following the manufacturer’s instructions. RNA quality was assessed with an Agilent RNA 6000 Nano Kit on the Bioanalyzer 2100 (Agilent Technologies, Santa Clara). Only samples with an RNA integrity number above 7.0 were selected for library construction.

sRNA libraries were prepared using the RealSeq-AC miRNA Library Kit for Illumina sequencing (Somagenics, Santa Cruz), starting with 150 ng total RNA and performing 16 PCR amplification cycles. Size selection of sRNA libraries to approximately 150-nt was achieved using SPRIselect Reagent (Beckman Coulter Life Sciences, Indianapolis) magnetic beads. RNA-seq (Smart-seq2; Illumina, San Diego) and nanoPARE libraries were prepared from 5 ng total RNA following the modified nanoPARE library preparation protocol (33) as described by Pokhrel et al. (34) with additional minor modifications: i) 13 PCR cycles were performed during the preamplification step and ii) the tagmentation enzyme (TDE1, Illumina) was optimized for mRNA libraries (0.5 µL or 1.0 µL) and nanoPARE libraries (1.25 µL or 1.5 µL). sRNA, mRNA, and nanoPARE libraries were quality-checked using the Bioanalyzer 2100 (Agilent Technologies, Santa Clara) before normalization and pooling for sequencing. Single-end reads of 51 cycles were generated for sRNA libraries using an Illumina NextSeq 2000, while single-end reads of 76 cycles were produced for nanoPARE and mRNA libraries on an Illumina NextSeq 550. Sequencing was performed at the University of Delaware DNA Sequencing and Genotyping Center.

### Single-Cell RNA Library Construction and Sequencing.

For single-cell RNA sequencing (scRNA-seq), we collected 0.4 mm premeiotic and 1.0 mm meiotic anthers. Samples were collected from WT at 20 °C and homozygous double mutant plants under restrictive (sterile at 18 °C) and permissive (fertile at 26 °C) conditions, with two anther replicates for each. We isolated and sorted single cells into three 96-well plates per anther following the FX-Cell protocol, and the libraries were prepared using a custom CEL-Seq2 library preparation as described by Marchant et al. (24). scRNA-Seq libraries were sequenced on a NovoSeq (Illumina) at Novogene Co. (Sacramento, CA) with paired-end 150 base-pair (bp) reads.

### Preprocessing and Mapping of sRNA-seq Data.

We used cutadapt v4.1 (35) to preprocess sRNA-seq reads by removing the first 5’ nucleotide (-u 1), trimming the 3’ adapter (-a TGGAATTCTCGGGTGCCAAGG), and filtering reads outside the length range of 19-nt to 25-nt (--minimum-length 19 to --maximum-length 25). For mapping preprocessed sRNA reads to the durum wheat genome assembly Svevo.v1 (36), we employed ShortStack v4.0.2 (37) with the following mapping parameters: --threads 20 --mmap u --mincov 1 --align_only --dicermin 20 --dicermax 24 --pad 75. After mapping, we analyzed the mapped reads to annotate phasiRNAs and miRNAs.

### Annotations and Analyses of phasiRNAs.

We utilized ShortStack v3.8.5 (37) to annotate loci producing phasiRNAs. Genuine phasiRNA loci were identified based on sRNA clusters with a “Phase Score” ≥ 40 and ≥ 2.0 RPM (reads per million). These loci are referred to as *PHAS* loci. The python toolkit “bioinfokit” (https://pypi.org/project/bioinfokit/) normalized the ShortStack-generated read count-matrix to RPM. RPM-normalized reads were used to determine phasiRNA accumulation peaks during meiosis progression and to visualize the 21-nt and 24-nt phasiRNA abundances using the R pheatmap v1.0.12 package (https://rdrr.io/cran/pheatmap/). Because the *dcl5* mutant lacks 24-nt phasiRNAs, normalizing to total mapped reads overestimates other small RNAs (e.g., miRNAs and 21-nt phasiRNAs). To correct this bias, we first computed the fold-change of miR159 in premeiotic and meiotic anthers—stages when 24-nt phasiRNAs peak—in *dcl5* versus WT. We then applied those stage-specific ratios to rescale 21-nt phasiRNA abundances before plotting with pheatmap. *PHAS* loci were categorized by phasiRNA length (21-nt or 24-nt) and peak accumulation stage (premeiotic, meiotic, or postmeiotic). We compiled information on *PHAS* loci coordinates, total abundance, peak accumulation stage, and length category (21-nt or 24-nt) of all annotated phasiRNA loci (Dataset S2).

### Annotation of miRNAs.

We used ShortStack v4.0.2 (37) to analyze alignment files for hairpin-derived sRNA loci. Predicted miRNA loci meeting the criteria for plant miRNA annotations (38) were selected for further analyses. Criteria for true positive miRNAs included: i) presence of a precursor, ii) detection of the miRNA* read, and iii) mapping of over 75% of reads to the miRNA/miRNA* duplex. Known miRNA families were annotated by aligning representative reads of miRNA loci (both miRNA and miRNA*) to monocot-derived miRNAs from miRBase release 22.1 (39, 40) using ncbi-blastn v2.11.0+ (41) with parameters: -strand both -task blastn-short -perc_identity 85 -word_size 7 -evalue 0.01 -num_alignments 1 -no_greedy -ungapped. We filtered homology hits and classified sRNA reads as known miRNAs based on criteria: i) ≤four mismatches and ii) ≤2-nt extension/reduction at 5’ or 3’ end.

MicroRNAs miR2118 and miR2275, which trigger 21-nt and 24-nt phasiRNAs, were identified at multiple loci in grass genomes, including polycistronic loci (42). Mapping mature miRNA and miRNA* to their true expression origins posed challenges due to these characteristics. Current mapping and miRNA annotation algorithms do not adequately resolve the origin of expressed miR2118 and miR2275. Consequently, we did not consider the precision criteria for miRNA/miRNA* production and the hairpin structure as described by Axtell and Meyers (38). Instead, we performed a homology search using the predicted MIR2118 and MIR2275 precursor sequences to identify all potential loci expressing these miRNAs. Loci without a predicted miRNA hairpin structure were annotated as MIR2118 and MIR2275 loci if associated sRNAs map to these regions. MicroRNAs lacking homology to known families were considered as candidate miRNAs if the mature miRNA length was 20 to 22 nt. Known and candidate miRNAs were summarized by family, with names based on genomic positions when multiple loci per family were present in the genome. Detailed information on coordinates, annotation, and abundance of all miRNA loci is compiled in Dataset S3.

### Analysis of RNA-seq and nanoPARE Data.

We used cutadapt v4.1 (35) to remove the adapters and trim low-quality nucleotides with a Phred quality score ≥ 20 (-q 20). RNA-seq and nanoPARE libraries were pooled for RNA transcript assembly, while nanoPARE libraries were separately analyzed to validate sRNA-directed cleavage sites of *PHAS* precursors.

To reconstruct transcripts expressed in anthers, RNA-seq and nanoPARE reads were mapped to the reference genome using HISAT2 v2.2.1 (43) with default parameters. Genome-mapped reads underwent de novo reference-guided transcript assembly using two assemblers: StringTie v2.2.1 (-c 1.5, -f 0.2, -s 20, and -m 150) (44, 45) and Scallop v0.10.5 (--min_transcript_length_increase 35) (46). Consensus transcripts were constructed using StringTie-merge with the following parameters: -F 1, -f 0.1, -m 200, and -g 1000 (44, 45). Transcript abundance was quantified using Stringtie (-e option), and a gene-level read-count matrix was generated using prepDE.py. The read-count matrix was normalized, and genes expressing transcripts at ≥3 cpm were retained after filtering with edgeR (47). Expressed transcript sequences were extracted using AGAT (https://github.com/NBISweden/AGAT). Assembled GTF and transcript files were used for annotation of coding and noncoding gene loci with TranSuite (48), and we identified genes coding protein involved in sRNA biogenesis and function (23). *PHAS* loci coordinates were used to retrieve 21-*PHAS* and 24-*PHAS* precursors using the intersect function from Bedtools (49). AGAT was employed to extract the *PHAS* precursors and used as input transcripts for nanoPARE analysis.

For posttranscriptional regulation analysis, nanoPARE reads were analyzed separately, focusing on miRNAs triggering *PHAS* transcript cleavage. Annotated *PHAS* precursors were used to predict and validate miR2118 and miR2275 targets with PAREsnip2 (50) using Fahlgren & Carrington targeting rules (51). All sRNAs were used to predict and validate the cleavage of premeiotic 24-*PHAS* precursors. Targets with *P*-value < 0.05 in categories 0 and 1 present across replicates were considered.

### Genome-Wide Analysis of nanoPARE, mRNA, and sRNA Reads Mapping to Conserved Motifs in Premeiotic and Meiotic 24-*PHAS* Loci.

To identify and analyze conserved motifs in premeiotic and meiotic 24-*PHAS* loci, we utilized the ShortStack-generated report to assess *PHAS* loci coordinates. Coordinates of these *PHAS* loci were extended using bedtools slop with a -b value of 1,000 and used to extract putative precursor sequences using bedtools getfasta (49). The extracted precursor sequences were analyzed with MEME v5.4.1 (52) to detect conserved nucleotide motifs previously described in premeiotic and meiotic/postmeiotic 24-*PHAS* loci (15, 17). This analysis allowed us to determine the precise sequences underlying the motifs, in the putative 24-*PHAS* transcripts, and to retrieve their genomic coordinates using ncbi-blastn v2.11.0+ (41) with the following parameters: -word_size 10 -perc_identity 100 -evalue 1e-10 -dust no -soft_masking false -max_hsps 1 -max_target_seqs 1 -ungapped. The resulting homology hits were converted into a Browser Extensible Data (BED) file format. We used bedtools v2.31.1 (49) with subcommands slop (with a -b value of 200) and getfasta (with the -s option for strandedness) to add 200 nucleotides of flanking regions upstream and downstream of the motif coordinates and extract their stranded sequences. To map nanoPARE, mRNA, and sRNA reads across these motifs and their flanking regions, we used Bowtie v1.2.3 (53). The resulting read coverage data were analyzed using bedtools genomecov (49) and we visualized the read coverage using the R package ggplot2 (54) generating a bar graph to illustrate the distribution of nanoPARE, mRNA, and sRNA reads in these regions.

### Analysis of scRNA-seq Data.

We processed the paired-end reads similarly to Nelms and Walbot (55). Briefly, the raw reads were demultiplexed based on cell-specific barcodes (*SI Appendix*, Table S4) using Fastq-Multx (56). The unique molecular identifier (UMI) sequences from read 1 were added to the read 2 sequence names and then filtered and trimmed with Fastp (parameters: -y -x -3 -f 6) (57). The clean reads were mapped to the durum wheat genome assembly Svevo.v1 (36) and the reference-guide de novo transcript assembly described above with HiSat2 (43), and UMIs quantified with SAMtools (58) and UMI-tools (59). Cell cycle heterogeneity has been shown to distort the clustering of cells; thus, all wheat homologs of previously identified maize cell cycle-regulated genes were removed (55). One multiplexed library from a sterile (18 °C) double mutant 0.4 mm anther was oversequenced based on significantly higher transcript counts and gene diversity compared to the two other replicates from the same anther and was removed from the dataset.

All statistical analyses referenced in the text and figures and all plots were performed in R/RStudio (https://posit.co) with the following packages: ggplot2, plyr, seurat, pheatmap, and paletteer. We performed normalization and cell type identification using Seurat 5.1 (60). We removed cells with fewer than 1,000 UMIs and cells with more than 20,000 distinct genes expressed due to the likely presence of doublets. We performed standard normalization methods in Seurat version 4.3 (61) using “LogNormalize” and a scale factor of 10,000. We identified wheat homologs of maize tapetal marker genes (*DCL5*, *MS23*, *MS32*, *bHLH122*, and *bHLH51*) from the literature (62) and found that the homologs of three of these genes (*DCL5*, *MS23*, and *bHLH51*) were highly expressed in select wheat cells. Cells expressing any of the wheat homologs (Dataset S4) of these three genes were used to identify wheat tapetal cells for further analysis.

## Supplementary Material

Appendix 01 (PDF)

Dataset S01 (XLSX)

Dataset S02 (XLSX)

Dataset S03 (XLSX)

Dataset S04 (XLSX)

## Data Availability

The complete set of raw sRNA-seq, RNA-seq, and nanoPARE reads was deposited in the Sequence Read Archive under accession number PRJNA1204935 (63).
