# Supplementary material for: DICER-LIKE 5 loss causes thermosensitive male sterility in durum wheat and reveals an AU-rich motif guiding 24-nt phasiRNA biogenesis
Source: Proc Natl Acad Sci U S A. 2025 Jul 30;122(31):e2504349122. doi: 10.1073/pnas.2504349122 (PMC12337324; doi:10.1073/pnas.2504349122)
Supplement: Supplementary file 1 — Appendix 01 (PDF) [file pnas.2504349122.sapp.pdf]

SUPPLEMENTAL FIGURES

Figure S1

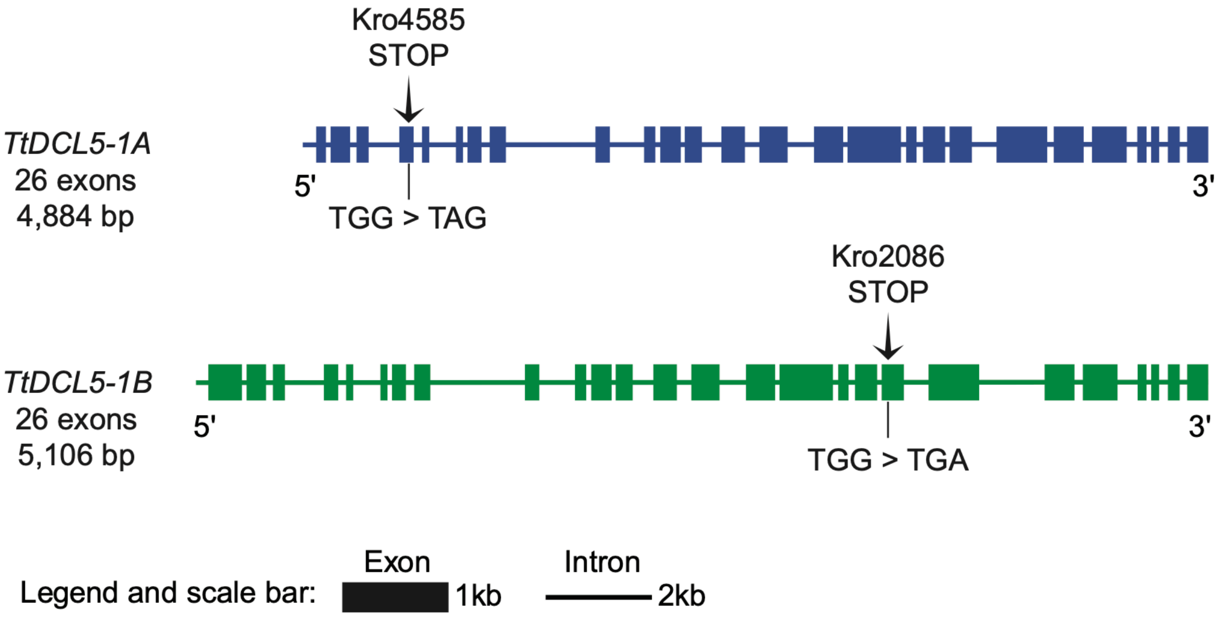

Fig. S1. *Mutations identified in TILLING lines utilized for the development of the* dcl5 *lines.* The TILLING lines, Kronos4585 (Kr4585) and Kronos2086 (Kr2086), identified from the Ensembl Plants database (28), were found with EMS-induced mutations in *TtDCL5-A1* and *TtDCL5-B1*, respectively. These mutations, identified as stop-gain variants (Variant IDs: Kronos4585.chr1A.289335805 in *TtDCL5-A1* and Kronos2086.chr1B.322412735 in *TtDCL5-B1*), lead to premature stop codons in both homeologs.

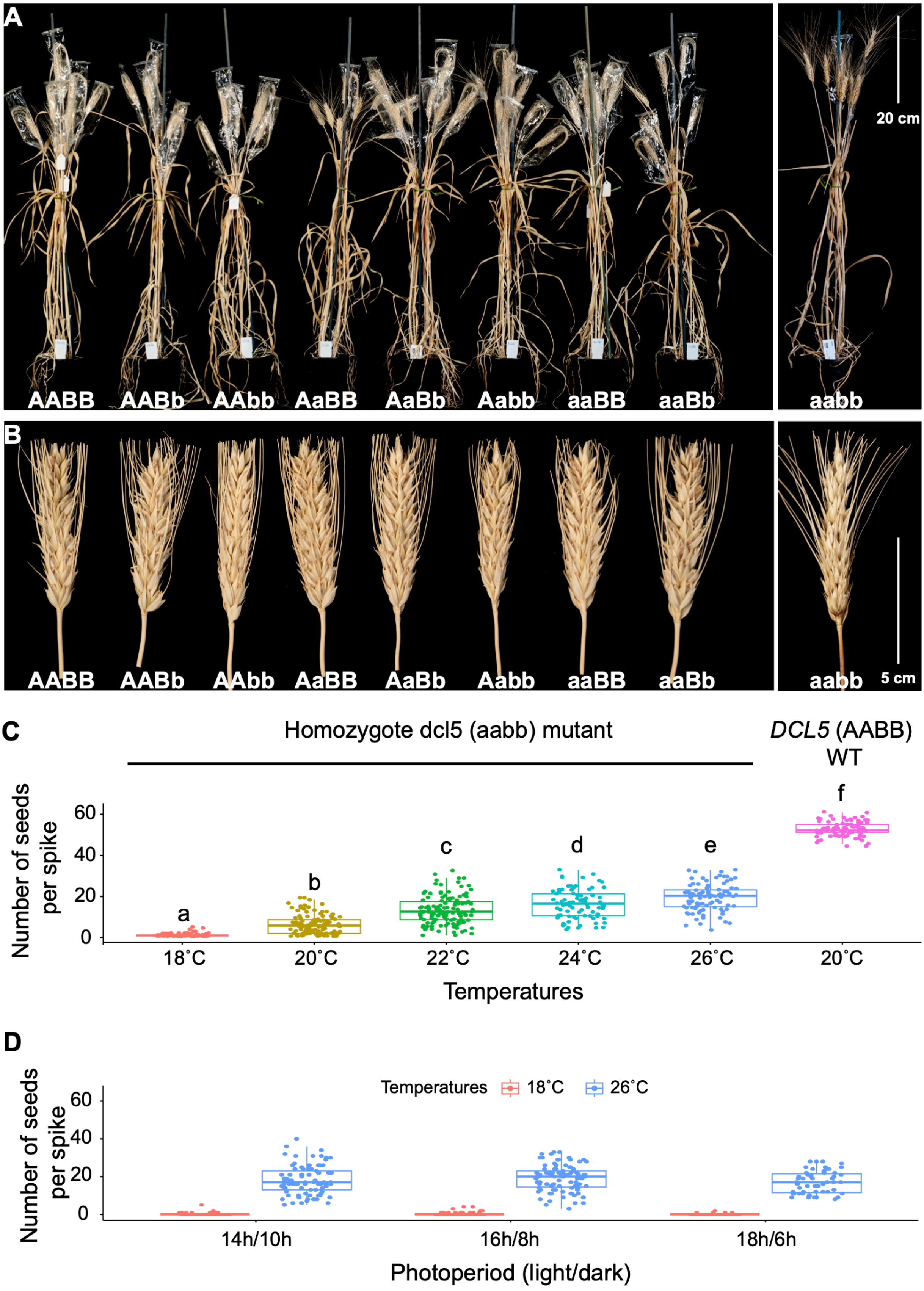
A
AABB
AABb
AAbb
AaBB
AaBb
Aabb
aaBB
aaBb
aabb
20 cm
B
AABB
AABb
AAbb
AaBB
AaBb
Aabb
aaBB
aaBb
aabb
5 cm
C
Homozygote dcl5 (aabb) mutant
DCL5 (AABB) WT
Number of seeds per spike
0
20
40
60
a
b
c
d
e
f
18˚C
20˚C
22˚C
24˚C
26˚C
20˚C
Temperatures
D
Temperatures
18˚C
26˚C
Number of seeds per spike
0
20
40
60
14h/10h
16h/8h
18h/6h
Photoperiod (light/dark)

Fig. S2. *No phenotypic differences were observed during the vegetative and reproductive development of mutants expressing one or more functional alleles of* dcl5 *in durum wheat.* Photos of whole plants (**A**) and spikes (**B**) from segregating siblings of *dcl5* durum wheat mutants compared to WT (AABB). (**C**) Number of seeds retrieved in spikes of the five most-productive tillers for the *dcl5* double mutant (aabb) grown at temperatures from 18˚C to 26˚C, incremented by 2˚C compared to WT (20˚C). These results show that the loss of *DCL5* produces male sterility under normal growth conditions (restrictive; 18˚C and 20˚C), while raising growth temperatures (permissive) gradually restores partial fertility and enables self-pollination at temperatures of 22˚C and higher. (**D**) Number of seeds per spike in the five most productive tillers of the *dcl5* double mutant (aabb) grown under shorter-day (14 h) and longer-day (18 h) photoperiods at temperatures promoting sterility (18 °C) or fertility (26 °C), as well as under control day-length (16 h) conditions.

Figure S3

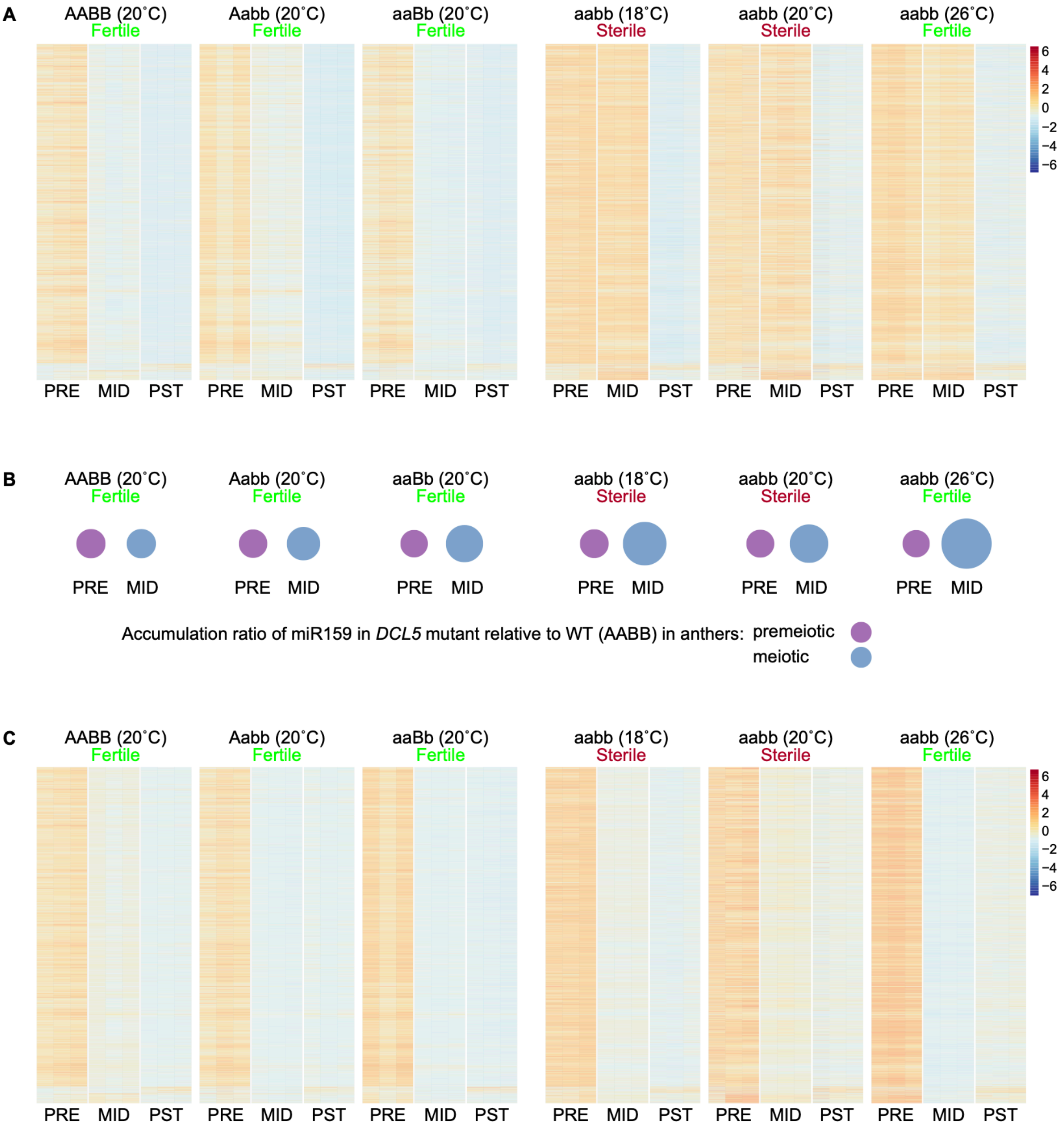

Fig. S3. *Loss of* DCL5 *does not affect the production of 21-nt phasiRNAs.*

(A) The heatmap illustrates the relative abundance of 21-nt phasiRNAs sequenced in triplicate samples of premeiotic (PRE), meiotic (MID), and postmeiotic (PST) anthers from the wildtype (AABB), the mutant expressing a single functional allele (Aabb and aabB), and the homozygous double mutant (aabb). We observed no depletion in the production of 21-nt phasiRNAs. In this panel, colors indicate relative abundance and are scaled by

row and thus do not reflect absolute or overall levels. (B) Bubble plot showing relative abundance of miR159 in premeiotic and meiotic anthers—when 24-nt phasiRNA levels peak—in *dcl5* mutants versus WT. This analysis was conducted to support a renormalization and alternative data visualization to panel A. (C) To better visualize the differences in abundances among the groups of phasiRNAs, the abundances of phasiRNAs from each locus (row) in panel A were normalized to the abundance of miR159 in that library, yielding a heatmap of adjusted 21-nt phasiRNA levels across genotypes.

Figure S4A

A

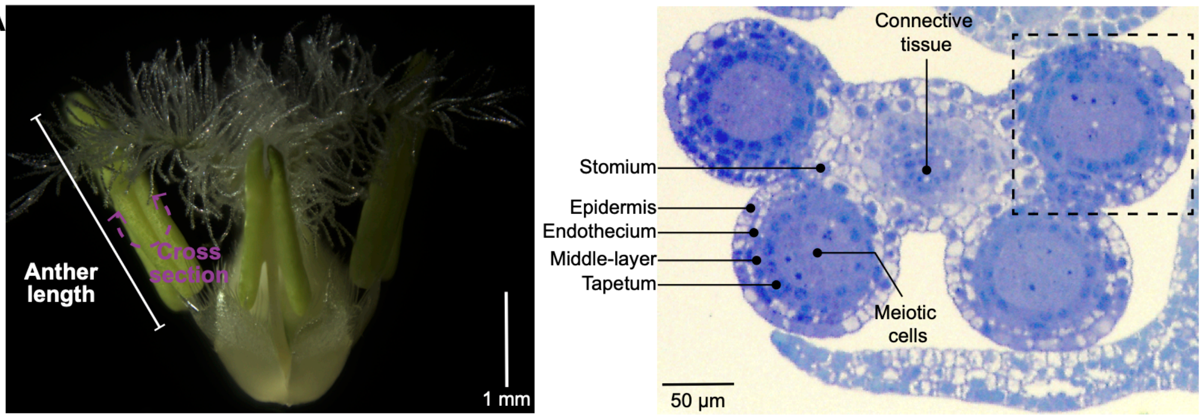
Anther
length
Cross
section
1 mm
Connective
tissue
Stomium
Epidermis
Endothecium
Middle-layer
Tapetum
Meiotic
cells
50 µm

Figure S4B

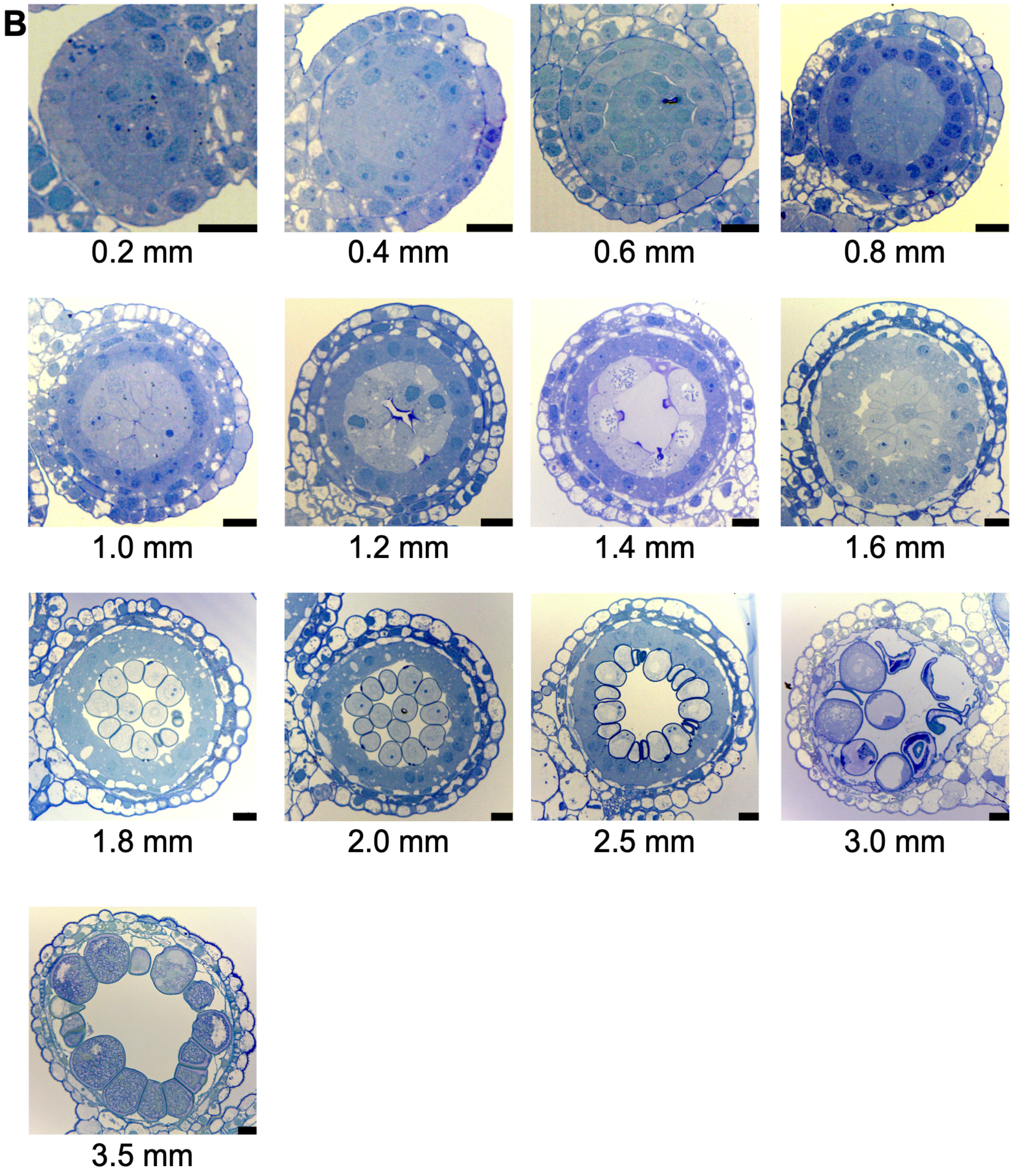
B
0.2 mm
0.4 mm
0.6 mm
0.8 mm
1.0 mm
1.2 mm
1.4 mm
1.6 mm
1.8 mm
2.0 mm
2.5 mm
3.0 mm
3.5 mm

Figure S4C

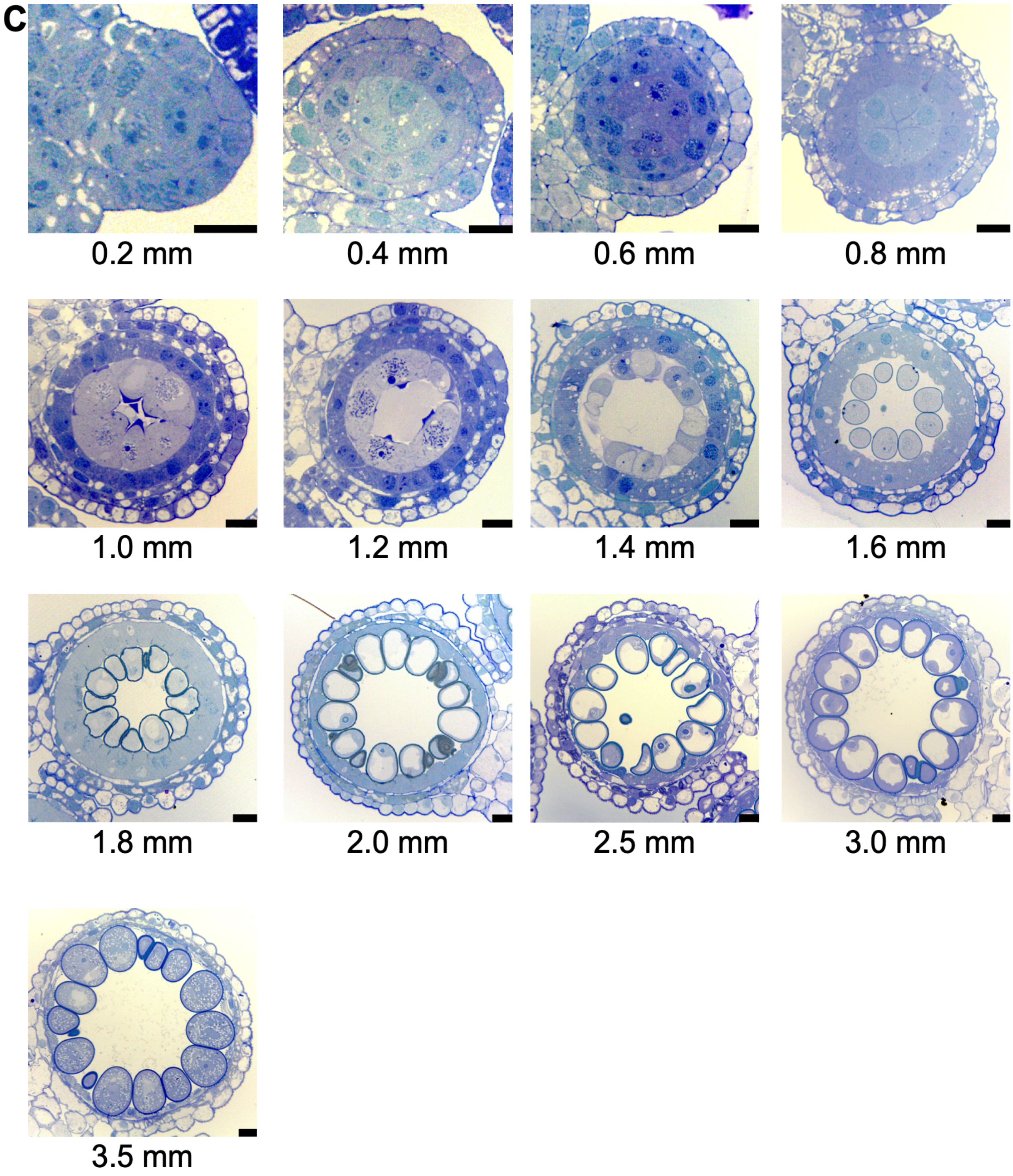
C
0.2 mm
0.4 mm
0.6 mm
0.8 mm
1.0 mm
1.2 mm
1.4 mm
1.6 mm
1.8 mm
2.0 mm
2.5 mm
3.0 mm
3.5 mm

Figure S4D

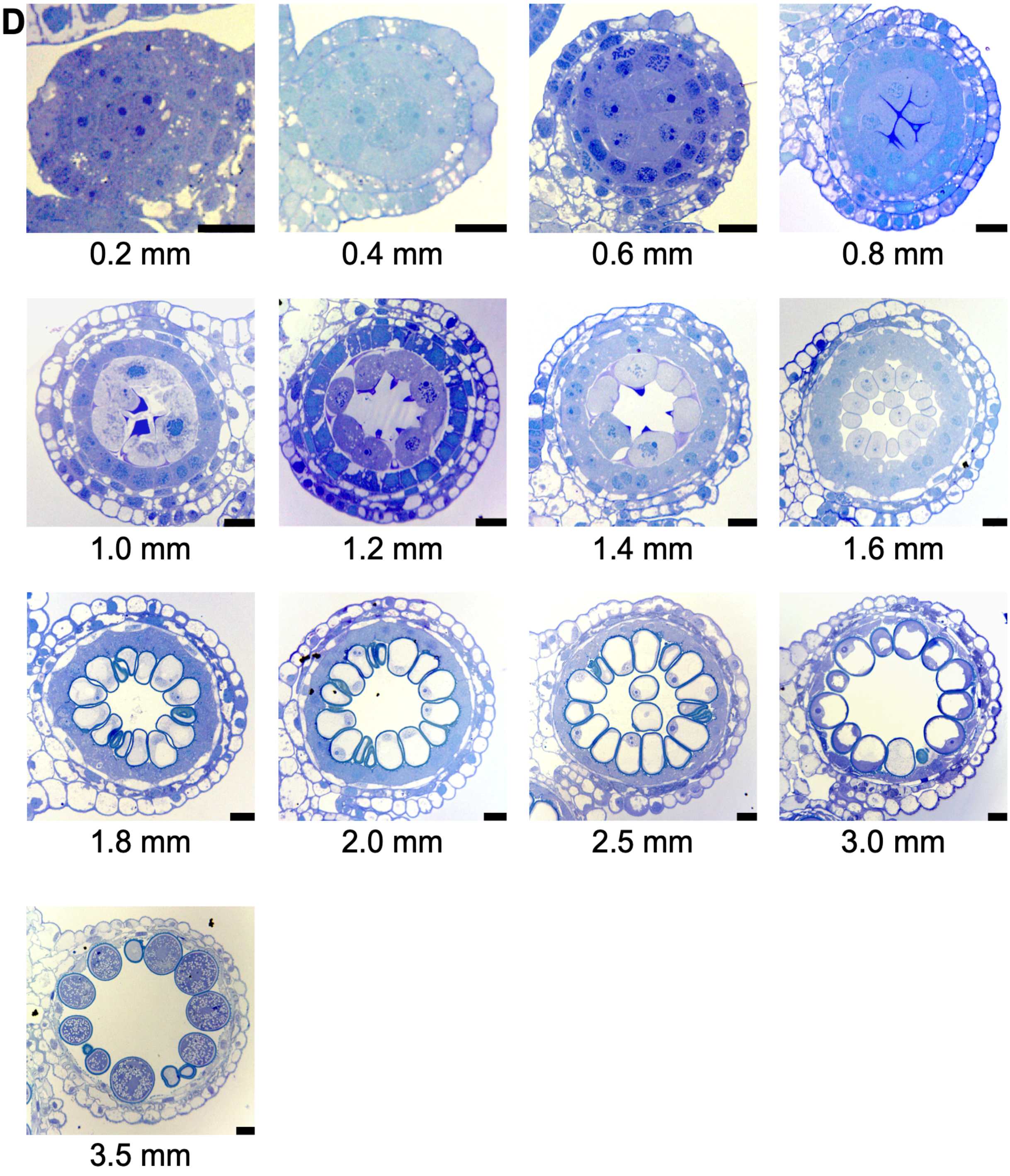
D
0.2 mm
0.4 mm
0.6 mm
0.8 mm
1.0 mm
1.2 mm
1.4 mm
1.6 mm
1.8 mm
2.0 mm
2.5 mm
3.0 mm
3.5 mm

Fig. S4. *Transverse sections of WT and* dcl5 *mutant anthers ranging in size from 0.2 mm anther to the stage of pollen maturation.*

(**A**) Cross-sections of anthers were taken from the middle portion of each anther (indicated by purple on the left), and images were captured from one lobe of the anther, as shown on the right. Anther length was determined as indicated on the left. Detailed anther sections of (**B**) sterile *dcl5* mutant, (**C**) fertile *dcl5* mutant, and (**D**) WT anthers. Anthers were fixed using a 2% paraformaldehyde:glutaraldehyde solution, embedded in Quetol resin, sectioned to 500 µm, and stained using Epoxy Tissue Stain. Scale bars = 20 µm.

Figure S5

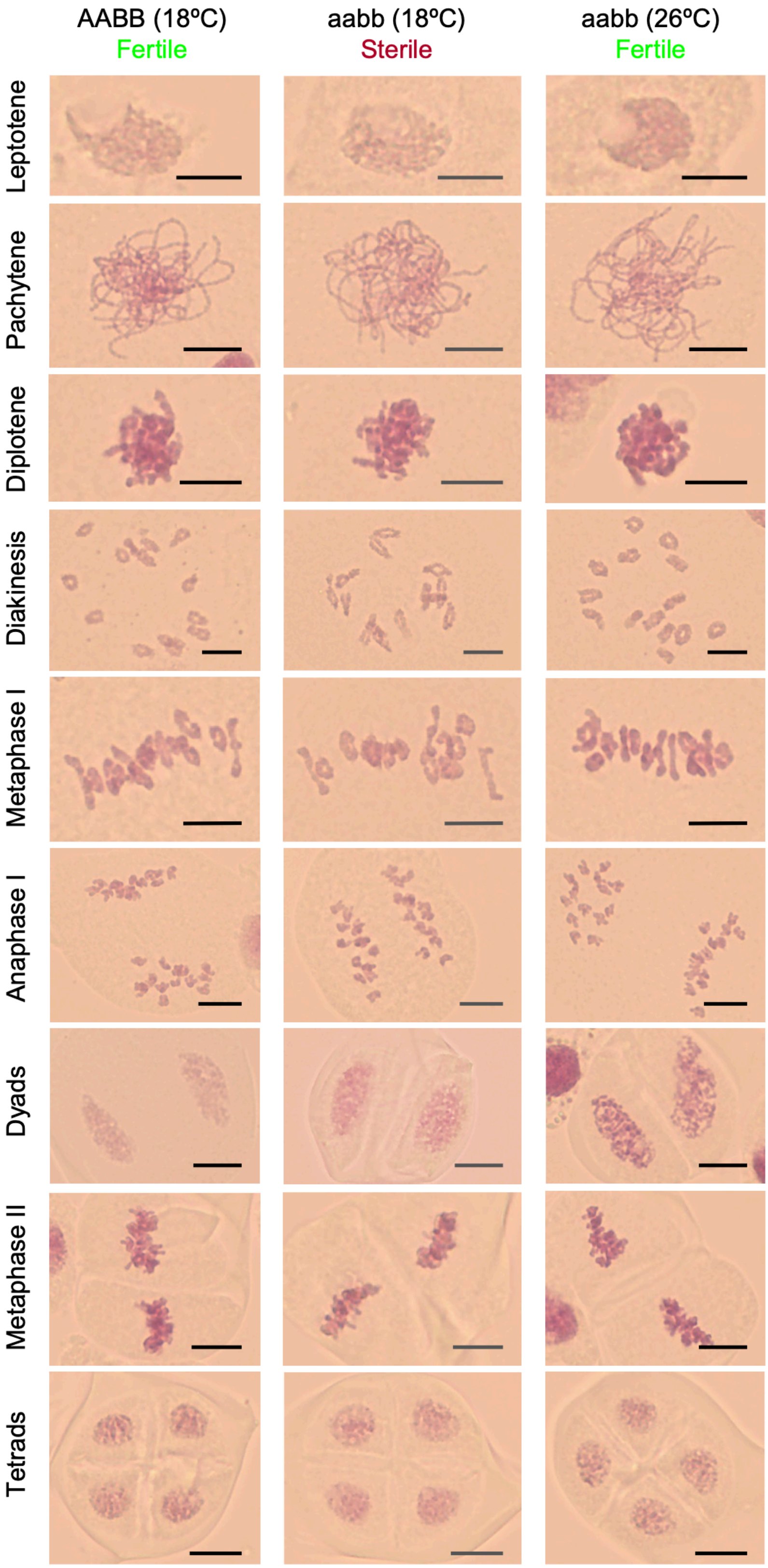

AABB (18ºC)
Fertile
aabb (18ºC)
Sterile
aabb (26ºC)
Fertile
Leptotene
Pachytene
Diplotene
Diakinesis
Metaphase I
Anaphase I
Dyads
Metaphase II
Tetrads

Fig. S5. *Loss of* DCL5 *does not affect meiosis.*

Male meiosis progresses normally in both WT and homozygous *dcl5* double mutants at sterile-inducing (18°C) and fertile-inducing (26°C) temperatures. Anthers from each genotype were fixed in ethanol:acetic acid (3:1) and stained with Schiff's reagent (Feulgen method) to visualize meiotic cells under light microscopy. Scale bar = 10 μm.

Figure S6

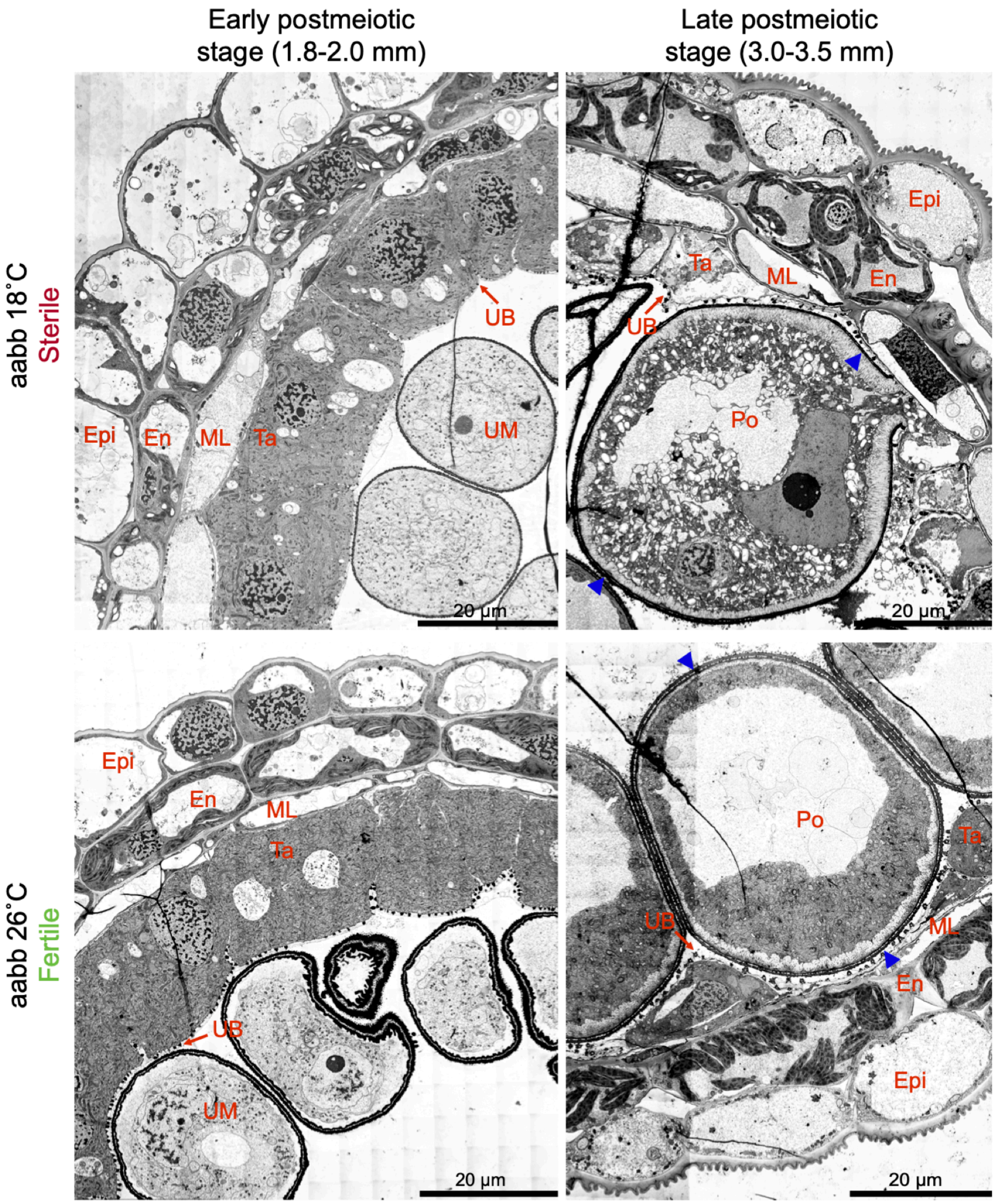

Fig. S6. *Anther ultrastructure showing dysfunctional pollen development.*

Transmission electron microscopy (TEM) was performed on anthers at two developmental stages of *dcl5* mutants grown under restrictive (top) and permissive

(bottom) conditions. At the early postmeiotic developmental stage (left), we observed less intimate contact at the interface between the tapetum (Ta), Ubisch bodies (UB), and uninucleate microspore (UM) in the anther developing under restrictive conditions (18°C; sterile) compared to the mutant developing under permissive conditions (26°C; fertile). At the pollen stage (right), differences in the thickness of pollen (Po) cell walls from the inner (thicker) and outer (thinner) sides were noted, indicated by blue triangles, in the anther developing under restrictive conditions, promoting cell wall rupture located at the pollen germination pore. Additionally, highly vacuolated middle layer (ML), endothecial (En), and epidermal (Ep) cells were observed in all sections.

Figure S7

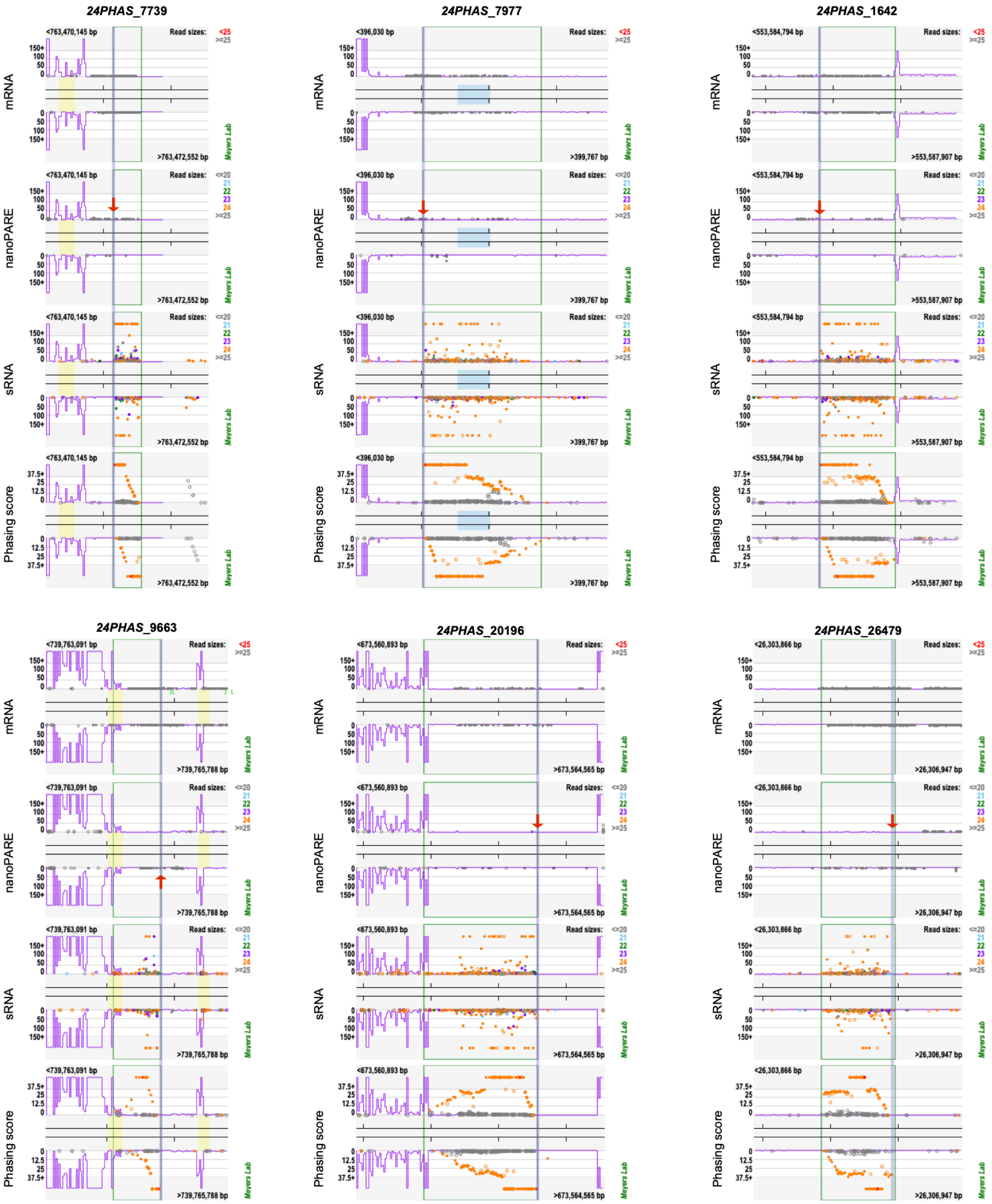

Fig. S7. *Premeiotic 24-*PHAS *loci precursors are independent of miRNA triggering and produce abundant, highly phased sRNAs.*

This figure illustrates mRNA, nanoPARE, and sRNA reads mapped to representative premeiotic 24-PHAS loci (each of three images stacked vertically), plus sRNA phasing scores (bottom image) for each locus. Shaded purple vertical lines across the four images designate the conserved premeiotic motif, while red arrows indicate motif midpoints (e.g. expected cleavage site) within the nanoPARE tracks. The tracks were generated by merging RNA libraries from WT and dcl5 lines and normalizing to reads per million (RPM) for mRNA (RP15M), nanoPARE (RP5M), and sRNA (RP10M). The phasing score is determined based on a ten-cycle window of sRNAs of 24 nt, with the score on the Y-axis; the red dot marks the window with the highest phasing score in the region, while other orange dots indicate windows in phase with the highest-scoring one. In this bottom image, filled dots represent perfectly phased windows, and hollow dots represent windows with -1/+1 mismatches. Grey hollow dots indicate out-of-phase windows, providing additional insights into the phasing landscape. Other details of the genome browser are described in Nakano et al. (2020).

Figure S8

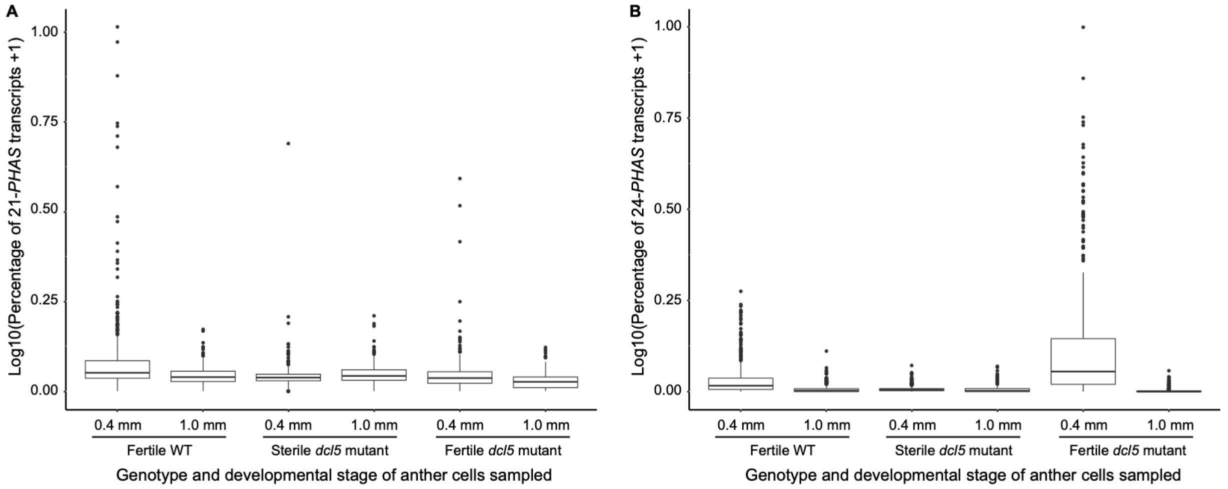

Fig. S8. *Fertile* dcl5 *cells accumulate abundant 24-*PHAS *loci.*

Proportion of 21-*PHAS* (**A**) and 24-*PHAS* (**B**) transcripts as a percentage of total transcript accumulation in individual cells from 0.4 mm and 1.0 mm anthers of WT and *dcl5* mutants under restrictive and permissive temperatures. The *y*-axis is expressed in log10(*x*+1).

Figure S9

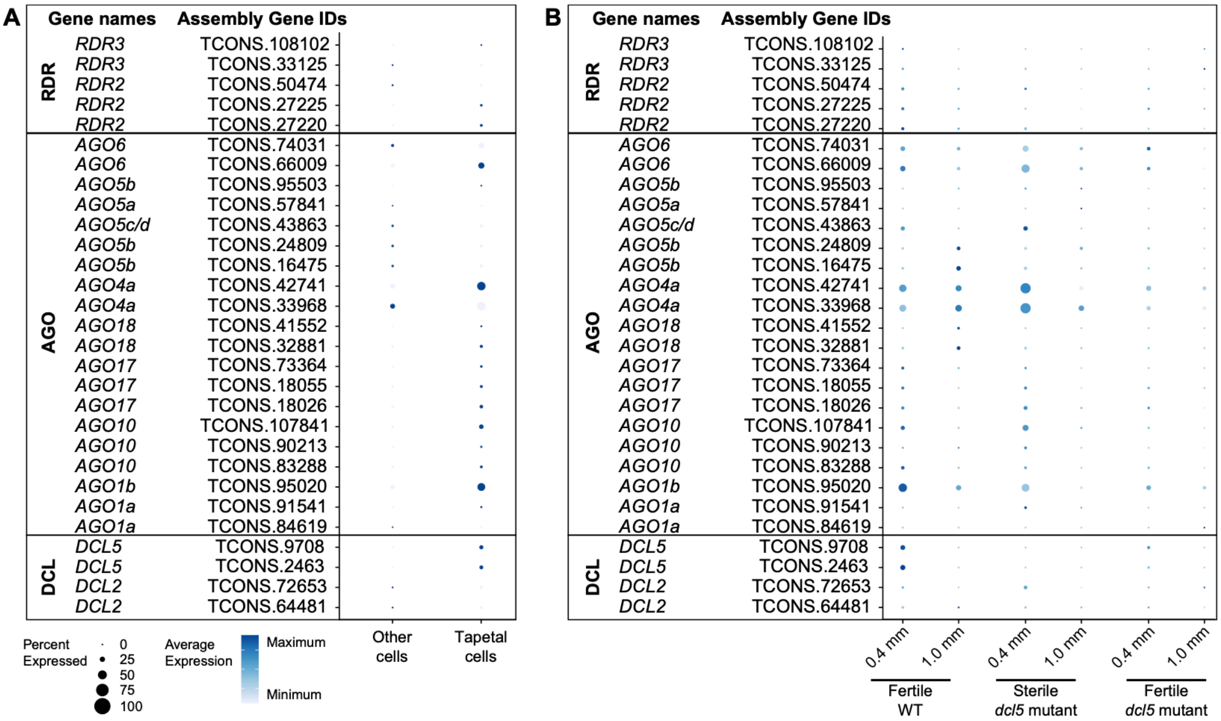

Fig. S9. *Specific* AGOs *are coexpressed with tapetum cells.*

Wheat homologs of maize genes known to regulate the expression of 24-*PHAS* transcripts and their processing into 24-nt phasiRNAs in tapetal cells were used as markers to subset putative tapetal cells and examine the coexpression of genes associated with the small RNA (sRNA) machinery. Bubble plots display the average expression levels of *DCL*, *RDR*, and *AGO* genes, as well as the percentage of cells expressing these genes in both putative tapetal and non-tapetal cells (**A**) and within tapetal cells across various genotypes and anther stages (**B**).

SUPPLEMENTAL DATASETS

Dataset S1. Gene-specific primer sequences for KASP genotyping of *TtDCL5-A1* and *TtDCL5-B1* mutant alleles in selected mutants.

Dataset S2. Coordinates and abundance of all PHAS loci detected in samples of premeiotic, meiotic, and postmeiotic anthers of WT (AABB), the *dcl5* mutant expressing a single functional allele (Aabb and aabB), and the homozygous *dcl5* double mutant (aabb).

Dataset S3. Coordinates and abundance of all miRNAs detected in samples of premeiotic, meiotic, and postmeiotic anthers of WT (AABB), the *dcl5* mutant expressing a single functional allele (Aabb and aabB), and the homozygous *dcl5* double mutant (aabb).

Dataset S4. Wheat homologs of maize tapetal marker genes (*DCL5*, *MS23*, *MS32*, *bHLH122*, and *bHLH51*) from the literature (62).
